# Filling gaps in PPAR-alpha signaling through comparative nutrigenomics analysis

**DOI:** 10.1186/1471-2164-10-596

**Published:** 2009-12-11

**Authors:** Duccio Cavalieri, Enrica Calura, Chiara Romualdi, Emmanuela Marchi, Marijana Radonjic, Ben Van Ommen, Michael Müller

**Affiliations:** 1 Department of Pharmacology, University of Firenze, Firenze, Italy; 2 Department of Biology, University of Padova, Padova, Italy; 3 TNO Quality of Life, BU Biosciences, the Netherlands; 4 Nutrigenomics Consortium, Top Institute Food and Nutrition, Wageningen, the Netherlands; 5 Division of Human Nutrition, Nutrition, Metabolism and Genomics group, Wageningen University, the Netherlands

## Abstract

**Background:**

The application of high-throughput genomic tools in nutrition research is a widespread practice. However, it is becoming increasingly clear that the outcome of individual expression studies is insufficient for the comprehensive understanding of such a complex field. Currently, the availability of the large amounts of expression data in public repositories has opened up new challenges on microarray data analyses. We have focused on PPARα, a ligand-activated transcription factor functioning as fatty acid sensor controlling the gene expression regulation of a large set of genes in various metabolic organs such as liver, small intestine or heart. The function of PPARα is strictly connected to the function of its target genes and, although many of these have already been identified, major elements of its physiological function remain to be uncovered. To further investigate the function of PPARα, we have applied a cross-species meta-analysis approach to integrate sixteen microarray datasets studying high fat diet and PPARα signal perturbations in different organisms.

**Results:**

We identified 164 genes (MDEGs) that were differentially expressed in a constant way in response to a high fat diet or to perturbations in PPARs signalling. In particular, we found five genes in yeast which were highly conserved and homologous of PPARα targets in mammals, potential candidates to be used as models for the equivalent mammalian genes. Moreover, a screening of the MDEGs for all known transcription factor binding sites and the comparison with a human genome-wide screening of Peroxisome Proliferating Response Elements (PPRE), enabled us to identify, 20 new potential candidate genes that show, both binding site, both change in expression in the condition studied. Lastly, we found a non random localization of the differentially expressed genes in the genome.

**Conclusion:**

The results presented are potentially of great interest to resume the currently available expression data, exploiting the power of *in silico *analysis filtered by evolutionary conservation. The analysis enabled us to indicate potential gene candidates that could fill in the gaps with regards to the signalling of PPARα and, moreover, the non-random localization of the differentially expressed genes in the genome, suggest that epigenetic mechanisms are of importance in the regulation of the transcription operated by PPARα.

## Background

The availability of public gene expression repositories, such as GEO [1] and ArrayExpress [2] has opened up new challenges on microarray data analyses, especially in the field of data integration and meta-analysis [3-6]. Meta-analysis, defined as the analysis of multiple gene expression datasets concerning a common biological problem, is performed to confirm, strengthen and complete the results obtained by single studies and to find common pathways altered in specific physiological or pathological conditions. Pivotal studies of this type have been performed on cancer [4] and have recently demonstrated their capability of retrieving much more relevant information than single experiment datasets [5,6]. In this study, we used a meta-analysis approach to investigate gene regulation and biological processes involved in response to high fat diet and under control of peroxisome-proliferator-activated receptor alpha (PPARα). As demonstrated by Bünger et al. the most of the nutritional science papers usually use transcriptome analysis merely as screening tool in order to focus the study on a single gene or on a pathway. On the one hand this type of approach simplifies the findings and their validations, but in the other hand miss the opportunity to observe the complete picture and infer the mechanism underlying the observations [7,8]. It is becoming increasingly clear that rather than reducing the information the challenge is now to fill gaps integrating information coming from genome-wide data, especially in nutrigenomics field.

Lipids serve as membrane constituents and supply and storage of energy. The related fatty acids are precursors of a wide range of bioactive molecules. They have the ability to regulate a wide variety of cellular processes through the induction of changes in gene expression. Indeed, fatty acids and some oxygenated derivates are ligands able to activate a class of transcription factors, called peroxisome-proliferator-activated receptors (PPARs), key regulators in energy storage and metabolism. PPARs (PPARα, PPARγ and PPARβ/δ) belong to a superfamily of nuclear hormone receptors that share a common action mechanism: the formation of heterodimers with the nuclear receptor RXR and the consequent binding to *cis*-element of promoter of a target gene.

PPARα is expressed primarily in metabolic tissues (brown adipose tissue, liver, kidney) but elevated levels are also present in the digestive (jejunum, ileum, colon, gall bladder) and cardiopulmonary (aorta, heart) systems, and plays a central role in almost all aspects of fatty acid catabolism in particular in the liver. Recent studies demonstrate that the role of PPARα is not limited only to metabolism but it also acts in many processes like inflammation [9], immunity [10], cardiovascular disease [11] and cancer [12], leading to the concept of an expanded activity of the nuclear receptor on more than one process. Lemay and Hwang in 2006 scanned the whole human genome using a PPRE matrix designed through the analysis of all known PPRE. The research was limited to conserved elements, as in space, evaluating neighbouring nucleotide, as in time, considering the sequence of different species, giving us a list of predicted PPAR targets [13]. Our study complemented the genome-wide analysis conducted to date by adding a meta-analysis performed across species of expression data related to PPARα signaling. Publicly available gene expression studies selected for our meta-analysis included experiments addressing molecular response to high fat diet, PPARα activation by various stimuli and gene expression in PPARα knock-out performed in a number of organisms and different array platforms. The comparison of gene expression across species (*Homo sapiens, Mus musculus, Rattus norvegicus and Saccharomyces cerevisiae*) is based on the known evolutionary conserved regulatory mechanism responsive to fatty acid-rich diet. In *S. cerevisiae *the homologous of mammalian PPARα-RXR is Pip2p-Oaf1p [14]. These two proteins form a heterodimer that activates the transcription of genes directly involved in peroxisome proliferation and fatty acid metabolism in response to a nutritional input [15]. Taking advantage of functional evolutionary constraints, comparison across species has potential to improve the comprehension of the biological mechanisms in response to fatty acid-rich diet suggesting novel candidate genes involved in PPARα signaling. Due to the evolutionary conservation, findings resulting from a cross-species analysis are expected to bring forward processes and genes that are most relevant for the studied condition. In addition, the discovery of novel PPARα targets homologous from yeast to human allows characterization of these genes in model organisms and extrapolation of the findings to the human situation.

In this study we provide a list of homolog genes subjected to comparable stimuli, showing significant changes in expression levels after changes in PPARα activity. Thus, taking advantage of all datasets available on PPAR signaling and high fat diet, our study offers a comprehensive overview of the key pathways and cellular processes regulated by PPARα. We identified a series of chromosomal regions in the mouse genome specifically enriched by PPARα related genes suggesting common regulatory mechanisms. Finally we provide a useful method and interesting information to identify new target genes integrating the results of our work on gene expression with those previously obtained by Lemay et Hwang on PPRE sequence [13].

## Methods

### Data collection

Array Express [16] and GEO [1] databases were used to select expression datasets suitable for the meta-analysis. Only datasets with CEL files for Affymetrix and raw intensity data for the other technologies have been taken into account. Additional datasets were selected to test the results of our work.

The data collection consisted of 16 datasets, that could be divided into three separate groups: (i) experiments on rat, mouse and human hepatocytes, where PPARα signaling is activated treating cells with WY14643, (ii) experiments in which PPARα signaling is completely inhibited using mouse PPARα knockout, (iii) experiments in which the organism under study were fed with high fat diet, comprehensive of the *Saccharomyces cerevisiae *dataset performed in our laboratory. On the whole, we included in the analysis 202 hybridizations (see Table 1 for details).

**Table 1:** Meta-analysis data collection.

PPARα signaling	n°	Reference	Dataset Accession Number GEO/AE	Org	Tissue	Technology
PPARα signaling activated by WY14643	1	[55]	GSE8302/E-GEOD-8302	Hs	Liver	Affymetrix
	
	2	[55]	GSE8302/E-GEOD-8302	Mm	Liver	Affymetrix
	
	3	[55]	GSE8302/E-GEOD-8302	Rn	Liver	Affymetrix

PPARα signaling repressed using PPARα knokout mice	4	[55]	GSE8290/E-GEOD-8290	Mm	Liver	Affymetrix
	
	5	[55]	GSE8291/E-GEOD-8291	Mm	Liver	Affymetrix
	
	6	[55]	GSE8292/E-GEOD-8292	Mm	Liver	Affymetrix
	
	7	[55]	GSE8295/E-GEOD-8295	Mm	Liver	Affymetrix

PPARα signaling activated by High fat diet	8	[56]	GSE8753/E-GEOD-8753	Mm	Liver	Affymetrix
	
	9	[57]	GSE6903/E-GEOD-6853	Mm	Liver	Affymetrix
	
	10	[58]	GSE8524/~	Mm	Liver	Affymetrix
	
	11	[59]	GSE1560/E-GEOD-1560	Mm	Aorta	Oligo Array
	
	12	[60]	GSE8700/E-GEOD-8700	Rn	Epididymal fat	Affymetrix
	
	13	[61]	~/E-MEXP-893	Mm	Colon mucosa	Affymetrix
	
	14	[62]	~/E-CBIL-24	Mm	Liver	Affymetrix
	
	15	[63]	GSE432/~	Mm	Liver	Oligo Array
	
	16	Marchi and Cavalieri in preparation	~/E-TABM-614	Sc	~	Oligo Array

Additional datasets were selected to test the results of our work. (see Table 2 for details). The validation set was composed of one dataset of *S.cerevisiae *with expression data of several knockout yeast for transcription factor involved in oleate response, and two mouse datasets with experiments belonging at three category of experimental design mentioned above.

**Table 2:** Validation data sets.

n°	PPARα signalling	Reference	Dataset Accession Number GEO/AE	Org	Tissue	Technology
1	PPARα signaling activated by WY14643 (PPARα WY14643-GSE8396)	[64]	GSE8396/E-GEOD-8396	Mm	Liver	Affymetrix

2	PPARα signaling repressed using PPARα knokout mice (PPARα KO1-GSE8396)	[64]	GSE8396/E-GEOD-8396	Mm	Liver	Affymetrix

3	PPARα signaling repressed using PPARα knokout mice (PPARα KO1-GSE8396)	[64]	GSE8396/E-GEOD-8396	Mm	Liver	Affymetrix

4	PPARα signaling activated by High fat diet (HFD-E-MEXP-1755)	[65]	~/E-MEXP-1755	Mm	Liver	Affymetrix

5	Oleate response repressed using knokout yeast of a transcription promoter (del_ADR1)	[50]	GSE5862/~	Sc	~	Oligo Array

6	Oleate response repressed using knokout yeast of a transcription promoter (del_PIP2)	[50]	GSE5862/~	Sc	~	Oligo Array

7	Oleate response repressed using knokout yeast of a transcription promoter (del_OAF1)	[50]	GSE5862/~	Sc	~	Oligo Array

8	Oleate response activated using knokout yeast of a transcription repressor (del_OAF3)	[50]	GSE5862/~	Sc	~	Oligo Array

9	Oleate response activated by High fat diet (oleate_vs_low_glucose)	[50]	GSE5862/~	Sc	~	Oligo Array

### Statistical analysis of microarray data

Gene expression of Affymetrix datasets were quantified and separately normalized using *rma *technique [17] and. EntrezGene Custom CDF file proposed by Dai et al. [18] was used to re-annotate Affymetrix probe sets in order to have an efficient and up-to-date genome annotation of array features. Raw data derived by oligo microarray were normalized using *lowess *algorithm exploiting MIDAW web tool [19]. In order to identify differentially expressed gene (hereafter DEGs) we performed SAM test [20], a moderated t-test with permutational approach. P-values and then Q-values (false discovery rate, FDR) were used to control test multiplicity, 0.05 was the chosen cut-off. Q-values for each gene were defined as: Q = (p*n)/*i*, where *p *is the *p-value *of the gene, *n *the total number of genes and *i *is the number of genes at or better than *p*. Statistical analyses was performed with R software http://www.r-project.org.

Unfortunately, not all the datasets contained sufficient numbers of biological replicates as required for powerful inference. Fold change cut-off, filtered by variance coefficient, was used to select DEGs in those datasets with less than 3 replicates per gene (GSE8302, GSE9291 and GSE9290).

### Pathways analysis on DEGs

Enrichment analysis on metabolic pathways was calculated for each dataset using Fisher exact test based on hypergeometric distribution with a *p-value *cutoff of 0.1. Similarity structure on metabolic enrichment characteristics across datasets was performed using cluster analysis. A Boolean matrix with pathways in rows and datasets in columns was generated, where matrix cells equal to 1 identified significant enrichment of a given pathway in a given dataset, and 0 otherwise [21,22]. Using TM4 [23] a hierarchical dendrogram based on Euclidean distance, average linkage and with bootstrap support was generated.

### Meta-analysis approach

Homologene database [24] was used to match DEG lists across different species. Mouse annotation was used as reference; thus, each gene has been converted to its correspondent *Mus musculus *HomologeneID.

Given the presence of some datasets without gene *p-value *(see previous paragraph for details), we decided to adopt as meta-analysis procedure the vote counting approach proposed by Rhodes and colleagues [3]. The vote counting approach allows the identification of a set of genes common to *j *of the *S *total number of datasets with *j *= 2...S. The idea was to compare the observed number of significant genes shared by at least *j *studies (observed gene enrichment) with the number of significant genes shared by at least *j *studies obtained by chance (random gene enrichment). The number *j *was defined through a permutational approach. Permutational steps were the following: i) Q-values of each dataset were randomly permutated so that genes in each signature (list of differentially expressed genes) changed randomly, but the number of genes in each signature remained the same, ii) the number of genes differentially expressed common to at least *j *datasets was calculated for *j *ranging from 2 to the total number of datasets, iii) step i) and ii) were repeated 1000 times, iv) average and empirical confidence intervals (at confidence level 95%) of the number of random gene enrichment for each *j *(across the 1000 simulations) were calculated. Then, we compared the observed number of genes shared by at least *j *studies with the confidence interval obtained through the permutational approach and choose those *j*s showing a significant difference between observed and random number of gene enrichment. Finally among these *j*s we selected the minimum *j *such that the ratio between the expected and observed number of genes shared was less than 10%. In our analysis the number of *j *leading to 4% of false positives was found to be equal to 6 [5]. Finally, meta-analysis approach produced a list of 164 genes, called MDEGs (Meta-analysis Differential Expressed Genes), given by the integration of the DEGs shared by at least 6 datasets (See Additional File 1 and 2 for details).

### Pathways analysis on MDEGs

An enrichment analysis, similar to that described in the previous paragraph, was applied on MDEGs. In this case, using hypergeometric distribution, the enrichment test set was represented by the MDEG list, while the reference set should be virtually generated according to the number *j *identified through the permutational approach described above. The virtual reference set for the hypergeometric distribution was obtained by selecting all the genes common in at least *j *platforms. In our analysis the reference set contained 15,463 genes. This new general approach, specifically adapted for meta-analysis enrichment analysis, showed several advantages, accurate results, faster and easier execution.

A gene ontology network was drawn and analysed by means of the BINGO plug-in [25] of Cytoscape software version 2.6.0 [26]. Statistical significance was calculated using hypergeometric test with an FDR cut off equal to 0.05.

### Gene signature validations

The validation set was divided by organism, we performed one validation with *S. cerevisiae *and one for mouse data. The differentially expressed genes of each dataset are filtered by the species specific MDEGs and matrix with genes in row and dataset in column were build. Using TM4 [23] a hierarchical dendrogram with bootstrap support was generated.

### Transcription factor binding site search

Over-represented putative transcription factor binding sites have been detected for the lists of differentially expressed genes with oPOSSUM web tool [27]. The default parameters suggested by the Authors have been used to find TFBSs in the genomic flanking regions 2000 bp upstream and downstream the transcription starting site of MDEGs. Two statistical measures (Z-score and Fisher exact one-tail probability) were calculated to determine which TFBS were significantly over-represented in the examined flanking regions. Z-score > 5 and Fisher *p-value *< 0.05 were used as significant cut-off thresholds.

### Chromosomal clustering

Following the approach proposed by Vogel et al. [28] we searched for correlations between chromosome location, regulation and function of genes. In order to find genes located in chromosome clusters along the genome, we used a bioinformatic tool called REEF [29]. The first analysis step used the distribution of MDEGs in the genome, performed with different parameters of "window width" and "window shift". The tool calculate the hypergeometric probability, taking into account the number of studied genes and the number of the genes in the genome contained in each window. Statistical significance was calculated using a cut off equal to 0.05.

## Results and discussion

### Meta-analysis of gene expression datasets

Our work aims at developing methodological and computational procedures for the study of metabolic pathways and conserved regulatory mechanisms underlying the fundamental biological response to high fat diet, with additional goal to suggest novel candidate genes belonging to PPARα signaling.

We used a cross-species meta-analysis approach for the integration, at the gene level, of sixteen transcriptional datasets from different organisms (human, mouse, rat and yeast) and experimental platforms (Affymetrix, single and double channels spotted oligonucleotides). The datasets included into the analysis were focused on either genetic or dietary perturbations.

**Figure 1:**
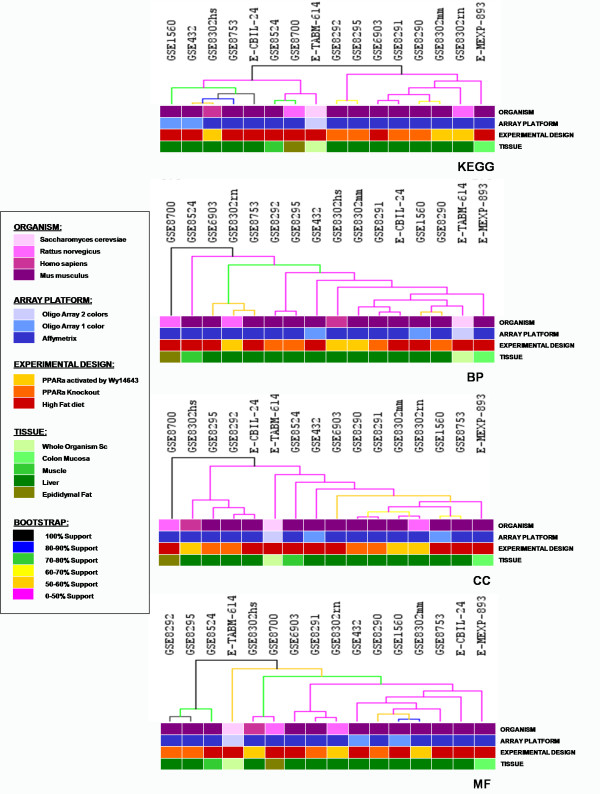
**Functional enrichment bootstrap support trees of datasets analyzed**. Biological Process (BP), KEGG's pathways (KEGG), Cellular Components (CC) and Molecular Functions (MF).

First, we evaluated the presence of possible trend in clustering due to organism, platform, tissue and experimental design variability characterising the sixteen datasets selected for the analysis. After inferential and enrichment analysis on each single dataset, functional similarities among studies have been performed through cluster analysis (see Methods for details). We expected that if some biases would be present in our analysis, datasets should be grouped according to the sources of variability. Figure 1 represents the dendrogram resulting from cluster analysis performed on GO and KEGG enriched classes. As shown in Figure 1, despite some differences, all the datasets seem to be highly similar, strictly comparable and not grouped according to the mentioned possible sources of variability. Thus, we proceeded with the meta-analysis approach in order to identify a set of marker genes highly relevant for the response to high fat diet and PPARα signaling. The clustering results support the evidence that dietary conditions modulating PPAR signaling and in general, high fat-low fat diet affect a coherent and evolutionary conserved core of genes, that overcomes the differences in gene expression associated to cell type, tissue and organ.

Analyzing the data intra and inter datasets, the meta-analysis approach allows us to find the most frequently deregulated genes in the tested condition.

Comparing the MDEG list with the DEG lists of datasets analyzed individually, we observe that the meta-analysis led us to identify a smaller number of total genes but biologically more strongly correlated to the studied condition, probably reducing the false positive genes and recovering true positive genes eliminated by a strict statistic at a single study level. A cross-species meta-analysis provides an added value to find conserved genes and for this reason is more reliable.

**Figure 2:**
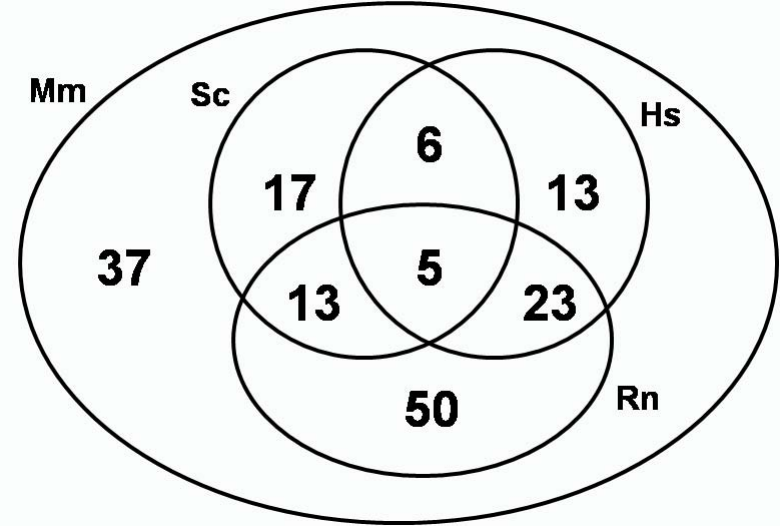
**Venn diagram of 164 MDEGs sharing among each organism**.

Meta-analysis approach identified 164 differentially expressed genes shared by at least 6 datasets (MDEGs) (for a complete list of MDEG see Additional File 1). The 164 MDEGs are selected with the consensus of at least 6 datasets regardless of the species. All the species are represented in the MDEG list but not all MDEGs are represented in all species excepted for mouse. This is due to the different number of organism specific experiments and the evolutionary distance between the species and the reference species. The Venn diagram represents the contribution of the four organisms in defining the MDEGs (Figure 2). The 100% of MDEGs (164 genes) are found in mice datasets, the 28.6% (47 genes) are found in human, the 25% (41 genes) in yeast and the 55.5% (91 genes) in rat datasets.

### Functional characterization of the MDEGs

**Figure 3:**
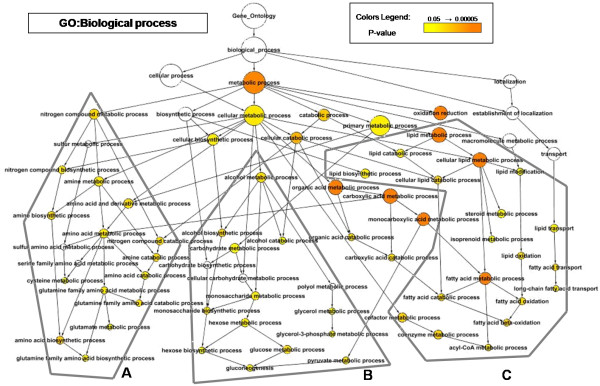
**Graphical representation of enriched biological process Gene Ontology category in MDEGs**. The network represented is a hierarchical network, the arrow from the element A to the element B signifies "the element B is part of the element A". The size of each circle is proportional to the number of gene contained in the category and circles are shaded based on significance. Red indicate P-value close to zero, yellow represent p-value close to cut-off threshold, white are not significant category. Biological Process (GO) category over-represented in MDEGs: **A) **amino-acid metabolism **B) **sugar metabolism **C) **lipid metabolism and transport.

**Figure 4:**
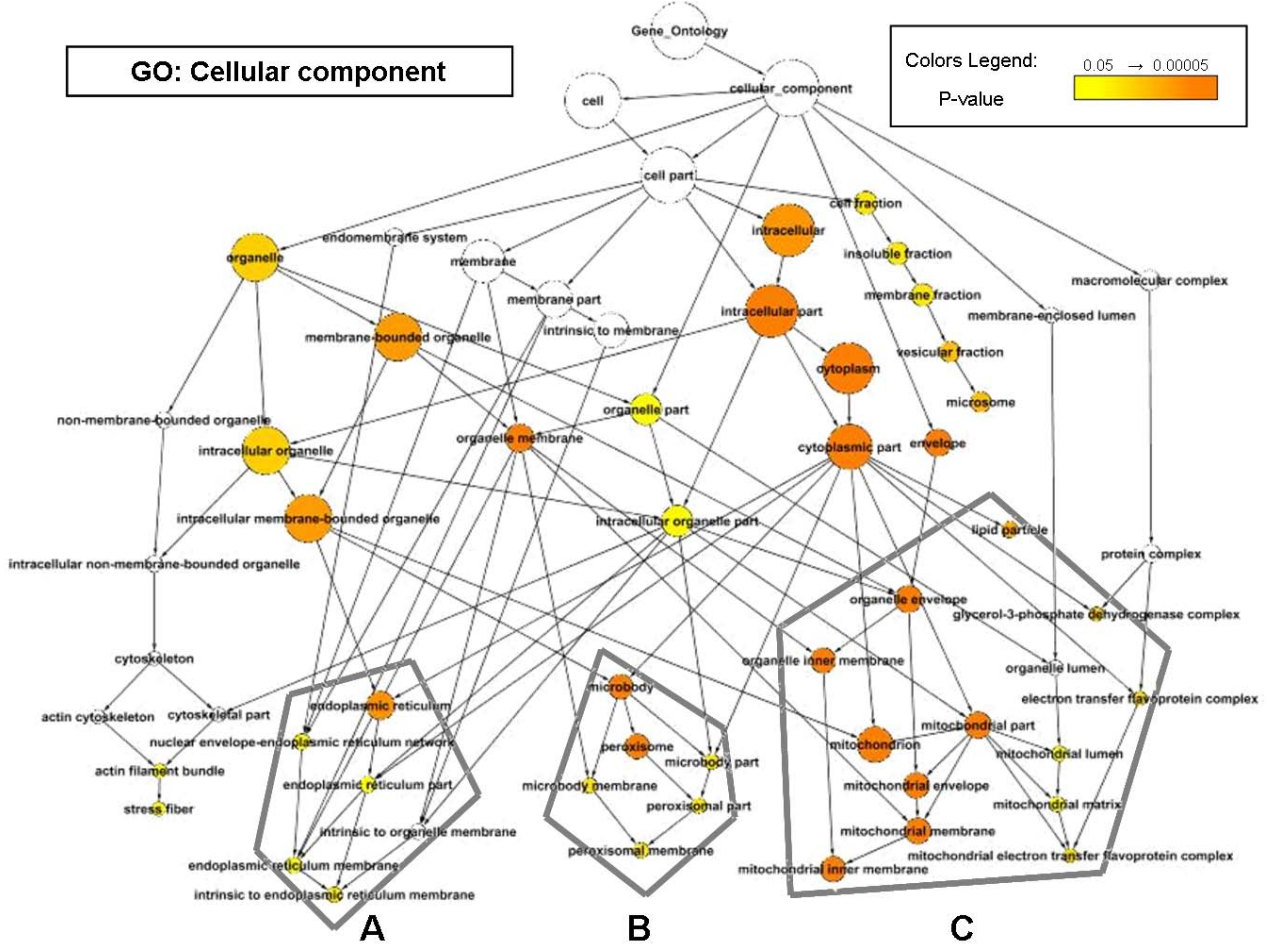
**Graphical representation of enriched cellular component Gene Ontology category in MDEGs**. Cellular Component (GO) category over-represented in MDEGs: **A) **endoplasmic reticulum **B) **peroxisome **C) **mitochondrion.

To study the identified set of 164 genes, we applied a network-based approach to describe the over-representation of GO categories in our pool of genes. The background organism used for this analysis was mouse, because it best represents all the genes highlighted in this study. This allows assessment of the reliability that our gene set reflects in the response to high fat diet and PPARα signaling as it is known by the literature (Figure 3 &4). For details on number of genes on each category, hypergeometric and FDR correction see Additional File 2.

Resulting biological process network (Figure 3) could be divided into three areas: i) group A, representing categories linked to amino acid metabolism, ii) group B, composed by carbohydrate metabolism, specifically related to monosaccharide metabolism and gluconeogenesis, and iii) group C, with categories associated to lipid metabolism and transport. These results give an overview of how PPARα modulates gene expression in order to regulate energy metabolisms.

The main over-represented cluster (cluster C Figure 3) was composed by lipid related categories, biogenesis, catabolism and transport of lipids. Fatty acid metabolism was one of the fundamental category of the network (Hsd17b4, Ehhadh, Dci and Acox1, Acadl and Acadvl), together with biosynthesis of lipids, represented by the enzyme to elongation of fatty acid (Elovl3, Elovl5, Elovl6) and Stearoyl-Coenzyme A desaturase 1 (Scd1) which catalyzes the rate limiting step in biosynthesis from unsaturated to saturated fatty acids. Moreover we found Me1 (Malic enzyme), which catalyzes the generation of NADPH required for fatty acid biosynthesis. Me1 and Scd1 are known as target genes of PPARα responsible for important parts of lipogenesis [30-32]. The activation of PPARα and fatty acid metabolism requires mobilization and transport within the cell and the engagement of various compartments of fatty acids. The categories of fatty acid transport in MDEGs were represented by Cpt1b, Cpt2 and Adfp. Cpt1b is a carnitine palmitoyltransferase enzyme, responsible for the oxidation of fatty acid allowing the translocation across the outer mitochondrial membrane and the starting of fatty acid oxidation. The carnitine palmitoyltransferase II (Cpt2) encodes for an enzyme embedded into inner mitochondrial membrane, that favours the reaction condensating coenzyme A with long-chain fatty acids facilitating the release from mitochondria. Cpt1b and Cpt2 are directly regulated by PPARα [33-38]. The intracellular transport of fatty acids to the nucleus and the nuclear receptor was represented in MDEGs by Fabp1, regulated by PPARα [39-41].

The representation of amino-acid metabolism pathway in GO network could be well explained by the interplay between anabolism and catabolism. In case of caloric restriction, amino acids are precursors for lipids, carbohydrates, and nucleic acids used as co-substrates and co-enzymes in the production of energy. On the contrary in case of dietary surplus, the potentially toxic nitrogen from amino acids has to be eliminated via transamination, deamination, and urea formation. Kersten et al. observed that in fasted mice the simultaneous increase in ketone body concentration and decrease in urea concentration are due to the action of a single factor, PPARα, which regulates the transcription of genes involved in the relative pathways, up-regulating fatty acid oxidation genes and downregulating the ureagenesis and ammonia detoxification.

PPARα regulates amino-acid metabolism through several genes, two of them, Cytosolic aspartate aminotransferase (Got1) and argininesuccinate lyase (Asl) were included in MDEGs [42,43].

Finally, Kersten et al. demonstrated that PPARα regulates carbohydrate metabolisms in particular by acting on gluconeogenesis [42] and governing hepatic glycerol metabolism [44].

All this evidence demonstrates that PPARα, despite its primary role in regulating the metabolism of fatty acids, acts as a master regulator of the rate of utilization of the various energy substrates in relation to food availability, and the identified network of biological process provides full details of all these aspects.

In addition to biological processes network, the cellular component network (Figure 4) highlights that the principal sites of activity during the response to high fat diet and PPARα signaling activation are the endoplasmatic reticulum (cluster A), the peroxisome (cluster B), and the mitochondrion (cluster C). The over-represented cellular components indicate that mitochondrion was the most over-represented scenario (26.8%; 44 genes) of the differential transcription regulation under the studied stimuli.

The results of pathway enrichment analysis are shown in Additional File 2. Several MDEGs belong to pathways related to high fat diet and 16 out of 164 MDEGs (10%) belonged to KEGG's Mouse PPARs signaling pathway (Acadl, Acaa1a, Acox1, Cpt1b, Cpt2, Cyp4a14, Cyp8b1, Fabp1, Hmgcs2, Me1, Scd1, Sorbs1, Acsl5, Angptl4, Ehhadh, Acsl3). Each of these 16 genes could be specifically connected to PPARα signal and not to the others different type of PPARs. Focusing on PPARα target genes in the whole set of 164 MDEGs, we found that the transcription of 42 genes (25.6%) was regulated by this nuclear receptor (Additional File 2). We also observed that only 11 of these 42 genes were characterized by functional PPRE and 5 genes have an *in silico *predicted PPRE.

### Evolutionary conserved markers of high-fat response

Cross-species analysis allows deciphering molecular complexity through evolutionary constrains. As expected mammalian organisms share the largest amount of genes (Figure 2), however, it is interesting to note that 41 yeast ORF (25%) were identified among MDEGs, each of these yeast genes has a homolog in mouse.

Looking at functions and processes linked to the yeast MDEGs, we observed that 7 out 41 genes, (17%; CAT2, FEN1, POT1, FOX2, YAT1, CRC1, SPS19) were directly involved in fatty acid metabolism and transport. CRC1, POT1, YAT1, FOX2 and SPS19 are targets of the transcription induced by Oaf1-Pip2 in yeast [45]. The 41 yeast genes identified by meta-analysis are localized both in mitochondrion (49%; 20 genes) and in peroxisome (12%; 5 genes). This strongly agrees with findings described in literature, stating that *S. cerevisiae *adapts to oleic acid as a sole carbon source inducing transcriptional modulation of both peroxisomal and mitochondrial function [46]. In addition to lipid metabolism, transcriptional reprogramming induced by oleic acid in yeast, as in the mammalian organisms, deregulates the amino acid metabolism (20%; 8 genes; EHD3, ARG4, CAT2, CYS3, CDC60, YAT1, GLN1, AAT2). Interestingly we discovered 5 yeast genes that are homologous of mammalian genes under control of PPARα. The 5 genes are ADP1 homologous of Abcg2 [47], AAT2 homologous of Got1 [42,43], ARG4 homologous of Asl [42], FOX2 homologous of Hsd17b4 [48,49] and YAT1 homologous of human CPT2 [37,38]. Interestingly only two genes Hsd17b4/FOX2 and CPT2/YAT1 appears to share the same transcriptional regulator PPARa in the mammal and Pip2p-Oaf1p in the yeast. This suggests FOX2 and YAT1 as central and evolutionary conserved response elements for the high fat diet response. Moreover these findings indicate that, either the regulatory structure remains to be completely elucidated, the other three genes could represent valid candidate genes for future investigations and that they could be used as model study for mammals genes exploiting the awesome benefits of yeast genetics.

### Gene signature validation

**Figure 5:**
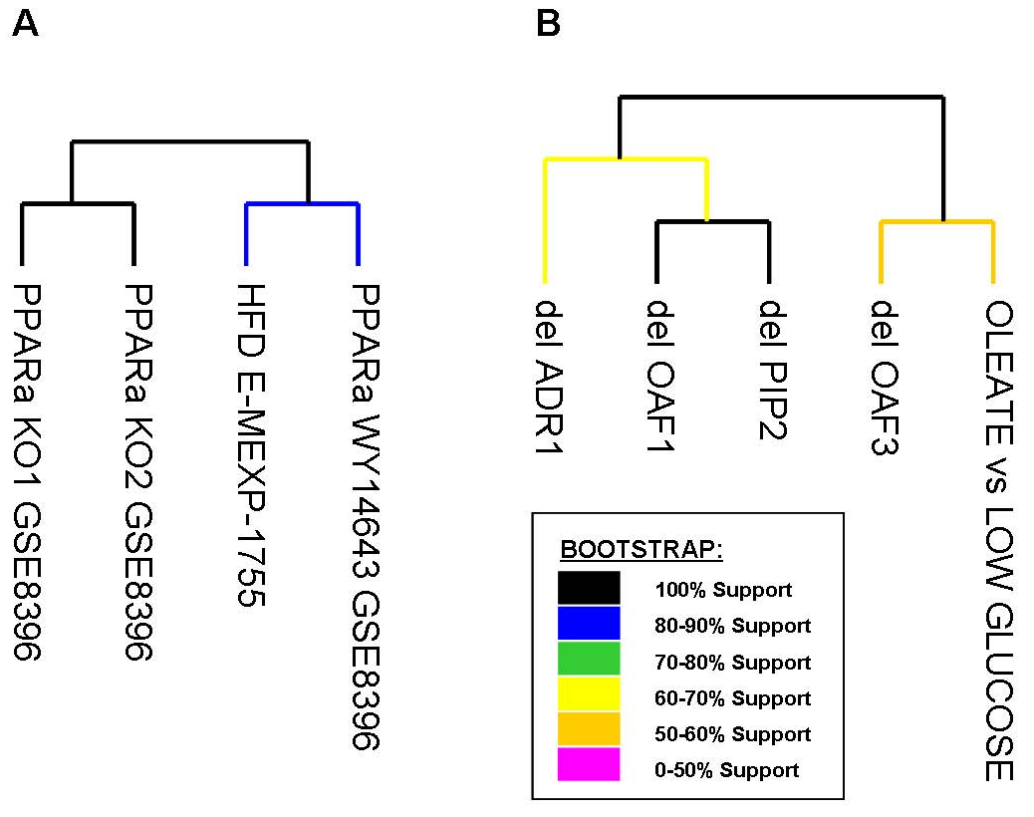
**Bootstrap support trees of validation datasets selected for MDEGs**. **A) **Yeast datasets **B) **Mouse datasets.

Further validation of our methodology and analyses came from the comparison of our gene list with several external expression dataset. We performed a validation for yeast, that have a gene signature composed of 41 MDEGs and a validation for mouse using all the 164 genes. The validation on yeast was performed using a dataset published in GEO database by Smith et al. [50]. Smith et al. (2007) performed the expression profiles of 4 transcription factor deletion strains (delta_OAF1, delta_PIP2, delta_ADR1 or delta_OAF3) in the presence of oleate and the expression profile of wild type strain in oleate versus low glucose diet. Our 41 yeast genes resulted as a subgroup of the pool of Smith, underlining the consistency of our list of genes in relation to the pathway of signal studied. In order to establish if our list is a useful gene signature to understand the activity of oleate response we selected the expression value of the 41 yeast MDEGs in each experiments of the Smith's dataset and we performed a cluster analysis. This analysis allows us to separate the datasets into two main groups. The first group contains the expression profiles of delta_OAF1, delta_PIP2, delta_ADR1 strains where the oleate-inducible transcription factors are deleted and therefore, the response to oleate diet is repressed. The second group is composed by oleate diet versus low glucose diet dataset, delta_OAF3 strain and our dataset used as a reference. The findings exactly mach to our expectations. As OAF3 is a repressor of oleate-induced transcription, delta_OAF3 strain has a behaviour similar to the activation of transcription induced by oleate. (Tree in Figure 5-B). The same procedure was applied to external expression dataset of *M. musculus*. The resultant tree of experiments, obtained clustering by similarity of expression the selected 164 mouse MDEGs, perfectly split the 4 experiments in 2 groups. In the first group we find experiment studying activation of PPARα signaling, in the second group experiment in which there is not PPARα. (Tree in Figure 5-A; Complete Matrix with expression values in Additional File 2).

The correspondence of the datasets separation to our expectations qualifies the identified genes as putative markers of the biological response to high fat diet.

### Transcription factor (TF) screening on MDEGs

Mining the literature we can find that Lemay and Hwang [13] have already performed a genome-wide screening of PPRE on human genome. Accordingly we compared the list of MDEG with the list of genes with PPRE provided by Lemay and Hwang, finding that 12 MDEGs have *in silico *predicted PPRE. We calculated the Fisher exact test p-value to study the overlap. As expected, because of the unbalanced numbers of the comparison, the p-value (p = 0.21) was not significant, although giving an indication of non randomness. However we are aware that the only presence of a PPRE does not necessarily result in a change in gene expression and vice versa many of the changes in gene expression we discover might be due to a not-direct interaction with PPARa or a combined action of more TFs.

With the aim of deeply investigate the presence of other TF binding sites, we accomplished a genome-wide screening of the region flanking the transcription starting site of all mouse MDEGs, searching binding site (TFBS) for all TFs contained in the Jaspar database [51]. The screening was performed using a tool called oPOSSUM [27]. Confirming and strengthening our previous results, the analysis shown that PPRE was the only TFBS over-represented in our pool of genes (see Additional File 2 for details). The target genes with predicted PPRE were 18 (Table 3). As expected the results of the two TF screening on MDEGs were partially different because of two different analysis pipelines [13]. Lemay and Hwang performed the screening using the whole human genome instead of the mouse one, they used a quite different sequence consensus for the PPAR binding site and different methods to assess a score to the findings. However the two analyses were similar for the purpose and, despite of the two different organisms used as reference, each method is developed taking into account species comparison. As consequence the human-mouse conserved elements have to be found and, in our opinion, no method is better than the other providing two complementary results.

**Table 3:** Table of MDEG with predicted Peroxisome Proliferator Response Element.

MDEG with oPOSSUM predicted PPRE in Mus musculus				MDEG with predicted PPRE by Lemay and Hwang in Homo sapiens			
**Gene Symbol**	**Entrez Gene ID**	**TFBS Position**	**Homolo** **Gene ID**	**Gene Symbol**	**Entrez Gene ID**	**TFBS Position**	**Reference**

				ACADVL	37	chr17:7060717-7060729	[38]
				
				G0S2	50486	chr1:206232580-206232592	[66]
				
				ELOVL3	83401	chr10:103974942-103974954	[67]
				
				TXNIP	10628	chr1:142924546-142924558	[55,68]
				
				SLC25A42	284439	chr19:19033468-19033480	-
				
				ACSL3	2181	chr2:223550325-223550337	-
				
				CREB3L3	84699	chr19:4104406-4104418	-

Lpcat3	14792	chr:6 124626752-124630751	14678	LPCAT3	10162	chr12:6996212-6996224	[69]

Hmgcs2	15360	chr:3 98363840-98367839	38066	HMGCS2	3158	chr1:120023667-120023679	[70-73]

Fabp1	14080	chr:6 71127471-71131470	1106	FABP1	2168	chr2:88270538-88270550	[39-41]

Ccnd1	12443	chr:7 144747221-144751220	1334	CCND1	595	chr11:69162605-69162617	[74]

Cpt1b	12895	chr:15 89251630-89255629	22548	CPT1B	1375	chr22:49311113-49311125	[34-36,74]

Nrp1	18186	chr:8 131243328-131247327					-
Nrp1	18186	chr:8 131241743-131245742					-
					
Sorbs1	20411	chr:19 40428334-40432333					-
Sorbs1	20411	chr:19 40429124-40433123					-
Sorbs1	20411	chr:19 40434177-40438176					-
					
Mmd	67468	chr:11 90063566-90067565					-
					
Igfbp2	16008	chr:1 72755699-72759698					-
					
Stk16	20872	chr:1 75092003-75096002					-
					
Rtn4	68585	chr:11 29616570-29620569					-
					
Hadh	15107	chr:3 131259199-131263198					-
					
Cxcl14	57266	chr:13 56304174-56308173					-
					
St5	76954	chr:7 109391843-109395842					-
					
Suclg1	56451	chr:6 73176158-73180157					-
					
Nnmt	18113	chr:9 48399563-48403562					-
					
Etfdh	66841	chr:3 79712646-79716645					[55]
					
Pnpla2	66853	chr:7 141303884-141307883					
Pnpla2	66853	chr:7 141304526-141308525					[55]
Pnpla2	66853	chr:7 141306941-141310940					

We believe that, despite the previous not significant p-value, the overlapping genes might represent an important set of response activators. This belief is strengthened by the fact that the overlap between the results of the two methods is composed by 5 genes (Lpcat3, Hmgcs2, Fabp1, Ccnd1, Cpt1b) all already known as target gene of PPARa (as shown in Table 3). This makes us confident that the remaining 20 genes, 13 of the oPOSSUM list and the 7 of Lemay and Hwang list, could be new candidate targets potentially interesting for further investigations.

### Co-localization of MDEGs across the genome

The regulatory mechanism at the basis of PPARα induction of transcription is not well understood. The presence of PPRE in the promoter and the simultaneous expression of the gene with the activation of the nuclear receptor are the best criteria required to confirm the regulation of a gene by PPARα. However, often we do not have both of these evidences to identify a target genes. Some genes without PPRE show fatty acid responsive changes in transcription and they seem to be under control of fatty acid regulation. In literature we find evidences supporting the idea that genes regulated by the same transcription factors and/or that sharing biological functions are co-localized in the genome [28].

Given the validity of our MDEGs, resulting from all the previous findings, for a better comprehension of the molecular mechanism underneath the high fat response, we explored MDEG genome arrangements across a mouse genome.

To increase the power of analysis we added to MDEG list the known PPARα target found in literature and not in our list. The analysis of the arrangement of the genes reveals a non-random chromosomal location, in particular, MDEGs are often co-localized to compose small group consisting of 2 to 5 genes (see Table in Additional File 2). Some of these clusters are very interesting for two reasons: (i) they contained genes that have been already demonstrated to be regulated by PPARα and in some cases the PPRE is known, and (ii) they contained genes with similar function and strongly correlated to the known activity of PPARα.

In our opinion, this finding is a further confirmation that the mechanism of transcription regulation operated by PPARα involve epigenetic processes. Indeed in literature we can find molecular and *in silico *confirmation of this hypothesis. Lemay and Wang, calculating functional enrichment of genes showing PPRE, have found that one of the most over-represented category was chromatin remodelling. In 1999 Xu et al. demonstrated that the recruitment of transcriptional machinery by nuclear receptors can occur directly or in response to chromatin remodelling, elicited by the dismissing of HDAC (Histone Deacetylase Complex), by ligand and by the recruitment of HAT complex (Histone Acetylase) [52]. Unfortunately this was not demonstrated specifically for PPARα. However, in recent years substantial effort has been invested in studies of chromatin remodelling complexes associated with transcription factors. In particular, Li et al. have shown that SMARCD1 is the molecular link between SWI/SNF chromatin remodelling complex and PPARα transcription factor. The recruitment of SMARCD1 to PPRE, mediated by PGC-1α, leads to a switch in chromatin structure to an active state [53]. In yeast, the connection between diet and chromatin remodelling is well studied. The nutritional status and chromatin state are correlated to health state and replicative life span, by mechanism involving sirtuin activation that regulates mitochondrial biogenesis through changing of the acetylation state of the transcriptional coactivator PGC-1α [54]. The fact that PGC-1α plays important role in epigenetic transcription regulation in both yeast and mammals as a physical link between PPRE bounded by PPARα and chromatin remodelling complex, suggest possible presence of the evolutionary conserved epigenetic regulatory mechanisms.

At the light of these findings, *in silico *analysis suggest that transcription factor induction and chromatin state seem to be the principal factors mediating the response to excess dietary fat. This probably allows PPARα to bind a PPRE and to regulate more than one gene at the same time.

## Conclusions

The proposed computational methods contribute towards the advances in integrative analyses of genomic data that still represent a major, and partially unresolved, computational issue. Through the selected strategy we were able to scan the expression data currently available and to suggest directions and new candidates to be investigated. This demonstrates the utility of the undertaken approach to exploit cross-species analysis and define gene signatures of the evolutionary conserved mechanisms as key elements to decipher the complexity of genome-wide data.

## Authors' contributions

DC and EC designed the experiment, performed the analyses, developed the method and wrote the paper, CR developed the method supported the analyses and wrote the paper, EM performed the yeast microarray hybridizations, MR discussed the results designed the experiment and corrected the paper, BVO corrected the paper and discussed the results, MM corrected the paper and discussed the results. All authors have read and approved the final manuscript.

## Supplementary Material

Additional file 1 The MDEGs list. Click here for file

Additional file 2 The file contain the complete output of BINGO, the KEGG enrichment table, the list of MDEGs know as target of PPARα, complete output of oPOSSUM, the table of co-localized genes. Click here for file
