# Supplementary material for: Filling gaps in PPAR-alpha signaling through comparative nutrigenomics analysis
Source: BMC Genomics. 2009 Dec 11;10:596. doi: 10.1186/1471-2164-10-596 (PMC2801700; doi:10.1186/1471-2164-10-596)
Supplement: Additional file 2 — The file contain the complete output of BINGO, the KEGG enrichment table, the list of MDEGs know as target of PPARα, complete output of oPOSSUM, the table of co-localized genes. [file 1471-2164-10-596-S2.PDF]

Supplementary Material Section 2: Statistical Output of BINGO

Biological Process

Parameters:

ontology: process
curator: GO
Selected ontology file : jar:file:c:\progra~1\cytosc~1.0\plugins\BiNGO.jar!/GO_Biological_Process
Overrepresentation
Selected statistical test : Hypergeometric test
Selected correction : Benjamini & Hochberg False Discovery Rate (FDR) correction
Selected significance level : 0.05
Testing option : Test cluster versus whole annotation
No annotations were retrieved for the following entities: 109254 107141 105387 231086 16855 20208 23945 56636 76954 21835 66917 72361 13101 68044 22750 219189 14964 235674 212531 18113 106564 337924 66968 432676 19683 75475

GO-ID	p-value	corr p-value	x	nX	N	Description
19752	2.06E-21	7.84E-19	36	391	138	carboxylic acid metabolic process
6082	2.06E-21	7.84E-19	36	391	138	organic acid metabolic process
32787	2.82E-19	7.15E-17	26	200	138	monocarboxylic acid metabolic process
44255	7.95E-18	1.22E-15	35	470	138	cellular lipid metabolic process
6631	7.98E-18	1.22E-15	22	146	138	fatty acid metabolic process
6629	1.35E-17	1.71E-15	37	542	138	lipid metabolic process
55114	4.82E-14	5.25E-12	31	482	138	oxidation reduction
8152	1.08E-08	1.03E-06	101	5330	138	metabolic process
44248	7.26E-07	6.15E-05	19	398	138	cellular catabolic process
8652	4.78E-06	3.64E-04	6	35	138	amino acid biosynthetic process
9056	6.25E-06	4.33E-04	20	504	138	catabolic process
6635	1.19E-05	7.53E-04	4	12	138	fatty acid beta-oxidation
51186	1.37E-05	8.05E-04	10	142	138	cofactor metabolic process
46165	1.71E-05	9.32E-04	5	26	138	alcohol biosynthetic process
6732	1.99E-05	1.01E-03	9	118	138	coenzyme metabolic process
44242	2.16E-05	1.03E-03	6	45	138	cellular lipid catabolic process
9062	3.17E-05	1.42E-03	4	15	138	fatty acid catabolic process
6807	3.35E-05	1.42E-03	13	264	138	nitrogen compound metabolic process
15908	4.02E-05	1.61E-03	3	6	138	fatty acid transport
6066	5.20E-05	1.98E-03	12	237	138	alcohol metabolic process
9309	6.23E-05	2.11E-03	6	54	138	amine biosynthetic process
34440	6.90E-05	2.11E-03	4	18	138	lipid oxidation
19395	6.90E-05	2.11E-03	4	18	138	fatty acid oxidation
9310	6.92E-05	2.11E-03	6	55	138	amine catabolic process
44270	6.92E-05	2.11E-03	6	55	138	nitrogen compound catabolic process
16054	8.66E-05	2.44E-03	4	19	138	organic acid catabolic process
46395	8.66E-05	2.44E-03	4	19	138	carboxylic acid catabolic process
9065	1.10E-04	3.00E-03	3	8	138	glutamine family amino acid catabolic process
6006	1.22E-04	3.21E-03	7	87	138	glucose metabolic process
6519	1.31E-04	3.22E-03	11	222	138	amino acid and derivative metabolic process
6090	1.31E-04	3.22E-03	4	21	138	pyruvate metabolic process
46364	1.59E-04	3.78E-03	4	22	138	monosaccharide biosynthetic process
44271	2.28E-04	5.26E-03	6	68	138	nitrogen compound biosynthetic process
6637	3.16E-04	7.09E-03	3	11	138	acyl-CoA metabolic process
9308	3.31E-04	7.13E-03	11	247	138	amine metabolic process
44237	3.43E-04	7.13E-03	82	4812	138	cellular metabolic process
6520	3.49E-04	7.13E-03	9	171	138	amino acid metabolic process
9063	3.55E-04	7.13E-03	5	48	138	amino acid catabolic process
9084	4.18E-04	8.16E-03	3	12	138	glutamine family amino acid biosynthetic process
9064	4.80E-04	8.74E-03	4	29	138	glutamine family amino acid metabolic process
15909	4.86E-04	8.74E-03	2	3	138	long-chain fatty acid transport
8610	4.91E-04	8.74E-03	10	218	138	lipid biosynthetic process
6724	4.93E-04	8.74E-03	7	109	138	lipid catabolic process

6869	5.16E-04	8.82E-03	6	79	138	lipid transport
19318	5.21E-04	8.82E-03	7	110	138	hexose metabolic process
5996	5.81E-04	9.62E-03	7	112	138	monosaccharide metabolic process
44249	7.82E-04	1.27E-02	18	610	138	cellular biosynthetic process
44238	8.65E-04	1.37E-02	79	4698	138	primary metabolic process
6072	9.64E-04	1.50E-02	2	4	138	glycerol-3-phosphate metabolic process
6094	1.23E-03	1.84E-02	3	17	138	gluconeogenesis
6071	1.23E-03	1.84E-02	3	17	138	glycerol metabolic process
8202	1.34E-03	1.96E-02	7	129	138	steroid metabolic process
5975	1.56E-03	2.24E-02	11	298	138	carbohydrate metabolic process
6536	1.59E-03	2.25E-02	2	5	138	glutamate metabolic process
30258	1.82E-03	2.52E-02	4	41	138	lipid modification
19319	2.01E-03	2.73E-02	3	20	138	hexose biosynthetic process
6534	2.37E-03	3.17E-02	2	6	138	cysteine metabolic process
16096	3.43E-03	4.51E-02	3	24	138	isoprenoid metabolic process
46164	3.51E-03	4.54E-02	4	49	138	alcohol catabolic process

Cellular component

Parameters

ontology: component
curator: GO
Selected ontology file : jar:file:c:\progra~1\cytosc~1.0\plugins\BiNGO.jar!/GO_Cellular_Component
Overrepresentation
Selected statistical test : Hypergeometric test
Selected correction : Benjamini & Hochberg False Discovery Rate (FDR) correction
Selected significance level : 0.05
Testing option : Test cluster versus whole annotation
No annotations were retrieved for the following entities: 107141 105387 231086 16855 20208 23945 76954 66917 109801 72361 13101 110208 68044 22750 12651 14964 12183 11564 235674 69748 106564 337924 432676 19683 193740 75475

GO-ID	p-value	corr p-value	x	nX	N	Description
5739	2.60E-19	3.25E-17	44	804	138	mitochondrion
44444	1.53E-18	9.55E-17	80	2829	138	cytoplasmic part
5737	1.14E-17	4.73E-16	106	5001	138	cytoplasm
19818	1.90E-15	4.74E-14	16	87	138	peroxisome
42579	1.90E-15	4.74E-14	16	87	138	microbody
44429	5.84E-11	1.22E-09	20	283	138	mitochondrial part
31966	9.41E-11	1.68E-09	18	228	138	mitochondrial membrane
5743	2.04E-10	3.18E-09	17	209	138	mitochondrial inner membrane
5740	3.07E-10	4.26E-09	18	245	138	mitochondrial envelope
31967	4.11E-10	4.73E-09	21	351	138	organelle envelope
31975	4.33E-10	4.73E-09	21	352	138	envelope
19866	4.54E-10	4.73E-09	17	220	138	organelle inner membrane
31090	1.59E-09	1.53E-08	24	497	138	organelle membrane
44424	4.39E-08	3.92E-07	115	7558	138	intracellular part
5783	3.30E-07	2.75E-06	24	657	138	endoplasmic reticulum
5622	5.68E-07	4.44E-06	115	7812	138	intracellular
43231	9.94E-07	7.31E-06	92	5625	138	intracellular membrane-bounded organelle
43227	1.05E-06	7.32E-06	92	5631	138	membrane-bounded organelle
5811	4.95E-06	3.26E-05	4	11	138	lipid particle
5792	2.13E-05	1.33E-04	9	134	138	microsome
42598	2.69E-05	1.60E-04	9	138	138	vesicular fraction
43229	1.16E-04	6.61E-04	93	6271	138	intracellular organelle
43226	1.22E-04	6.61E-04	93	6277	138	organelle
9331	1.28E-04	6.67E-04	2	2	138	glycerol-3-phosphate dehydrogenase complex
17133	3.82E-04	1.83E-03	2	3	138	mitochondrial electron transfer flavoprotein complex
45251	3.82E-04	1.83E-03	2	3	138	electron transfer flavoprotein complex
31903	7.21E-04	3.22E-03	3	16	138	microbody membrane
5778	7.21E-04	3.22E-03	3	16	138	peroxisomal membrane

267	1.21E-03	4.91E-03	13	430	138	cell fraction
44438	1.22E-03	4.91E-03	3	19	138	microbody part
44439	1.22E-03	4.91E-03	3	19	138	peroxisomal part
5624	1.48E-03	5.79E-03	12	386	138	membrane fraction
5626	1.76E-03	6.67E-03	12	394	138	insoluble fraction
31227	2.44E-03	8.96E-03	3	24	138	intrinsic to endoplasmic reticulum membrane
5789	7.80E-03	2.78E-02	4	69	138	endoplasmic reticulum membrane
42175	9.48E-03	3.29E-02	4	73	138	nuclear envelope-endoplasmic reticulum network
44446	1.19E-02	3.91E-02	31	1818	138	intracellular organelle part
44432	1.19E-02	3.91E-02	4	78	138	endoplasmic reticulum part
44422	1.27E-02	4.03E-02	31	1827	138	organelle part
5759	1.35E-02	4.03E-02	3	44	138	mitochondrial matrix
31980	1.35E-02	4.03E-02	3	44	138	mitochondrial lumen
32432	1.39E-02	4.03E-02	2	16	138	actin filament bundle
1725	1.39E-02	4.03E-02	2	16	138	stress fiber

Supplementary Material Section 3: KEGG enrichment table

The first column is the code and the name of the pathway, the second the number of MDEGs belonging to it, the third column is the P-value calculated with hypergeometric distribution and in the last the p-value corrected for multiple testing. Threshold value has selected as Q-value < 0.01.

**Pathway**	**N°**	**P-value**	**Q-value**
mmu03320:PPAR signaling pathway	16	0,000000	0,000000
mmu00071:Fatty acid metabolism	20	0,000000	0,000000
mmu00280:Valine, leucine and isoleucine degradation	12	0,000000	0,000000
mmu00640:Propanoate metabolism	6	0,000000	0,000000
mmu00062:Fatty acid elongation in mitochondria	4	0,00000	0,000000
mmu00650:Butanoate metabolism	7	0,000000	0,000000
mmu01040:Polyunsaturated fatty acid biosynthesis	5	0,000000	0,000000
mmu00120:Bile acid biosynthesis	6	0,000000	0,000000
mmu00310:Lysine degradation	6	0,000000	0,000002
mmu00380:Tryptophan metabolism	6	0,000001	0,000007
mmu00620:Pyruvate metabolism	5	0,000002	0,000017
mmu00410:beta-Alanine metabolism	4	0,000002	0,000018
mmu00272:Cysteine metabolism	3	0,000008	0,000056
mmu00980:Metabolism of xenobiotics by cytochrome P450	5	0,000010	0,000063
mmu00252:Alanine and aspartate metabolism	4	0,000015	0,000089
mmu00592:alpha-Linolenic acid metabolism	3	0,000020	0,000116
mmu00591:Linoleic acid metabolism	4	0,000027	0,000150
mmu00072:Synthesis and degradation of ketone bodies	2	0,000040	0,000207
mmu00590:Arachidonic acid metabolism	5	0,000049	0,000242
mmu00053:Ascorbate and aldarate metabolism	2	0,000063	0,000298
mmu00220:Urea cycle and metabolism of amino groups	3	0,000077	0,000333
mmu04920:Adipocytokine signaling pathway	5	0,000090	0,000373
mmu00550:Peptidoglycan biosynthesis	1	0,000112	0,000431
mmu00010:Glycolysis / Gluconeogenesis	4	0,000122	0,000436
mmu00330:Arginine and proline metabolism	3	0,000205	0,000709
mmu00564:Glycerophospholipid metabolism	4	0,000338	0,001136
mmu00480:Glutathione metabolism	3	0,000551	0,001791
mmu00770:Pantothenate and CoA biosynthesis	2	0,000592	0,001867
mmu00950:Alkaloid biosynthesis I	1	0,000661	0,002023
mmu00910:Nitrogen metabolism	2	0,000850	0,002525
mmu00260:Glycine, serine and threonine metabolism	3	0,000909	0,002627
mmu00710:Carbon fixation	2	0,001353	0,003804
mmu00350:Tyrosine metabolism	3	0,001525	0,004173
mmu00520:Nucleotide sugars metabolism	1	0,001630	0,004348
mmu00632:Benzoate degradation via CoA ligation	2	0,002012	0,005231
mmu00030:Pentose phosphate pathway	2	0,002269	0,005487
mmu00251:Glutamate metabolism	2	0,002269	0,005618
mmu00052:Galactose metabolism	2	0,003157	0,007297
mmu00642:Ethylbenzene degradation	1	0,003832	0,008663
mmu00150:Androgen and estrogen metabolism	2	0,004232	0,009366

Supplementary Material Section 4: List of MDEGs Know as target of PPARa

Homolo GeneID	Entrez GeneID	Gene Symbol	Sp.	Presence of PPRE/ORE	Change in expression PPARa/PiP2p dependent
41654	66968	Plin5	Mm		[1]
2140	19299	Abcd3	Mm		[2]
55852	26357	Abcg2	Mm		[2]
37498	11363	Acadl	Mm		[3, 4]
5	11370	Acadvl	Mm		[3]
22686	26897	Acot1	Mm		[5]
25661	171210	Acot2	Mm		[5]
38299	50681	Acox1	Rn	[6]	[7]
	11430	Acox1	Mm	[8]	[9-15]
872	11520	Adfp	Mm		[16]
10755	57875	Angptl4	Mm		[17]
47927	25080	Apoa4	Rn		[18]
32	109900	Asl	Mm		[19]
1334	12443	Ccnd1	Mm		[20]
22548	1375	CPT1B	Hs	[21, 22]	[14, 23]
77	1376	CPT2	Hs	[24]	[3]
	851285	YAT1	Sc	[25]	[25]
331	854267	CRC1	Sc	[25]	[25]
111391	13112	Cyp3a11	Mm		[26]
69011	13119	Cyp4a14	Mm		[20, 26]
3233	1582	CYP8B1	Hs	[27]	[27]
37554	13177	Dci	Mm		[18]
1486	171142	Ehhadh	Rn	[28]	[29]
47960	12686	Elovl3	Mm		[30]
3275	66841	Etfdh	Mm	Predicted [31]	[31]
1503	110826	Etfb	Mm	Predicted [31]	[31]
1106	14080	Fabp1	Mm	[32]	[15, 33]
10428	56636	Fgf21	Mm		[34, 35]
7326	14373	G0s2	Mm	[36]	[36]
1571	14718	Got1	Mm		[19, 37]
38066	15360	Hmgcs2	Mm		[38, 39]
	24450	Hmgcs2	Rn	[40]	[39, 41]
68471	15483	Hsd11b1	Mm		[18, 42]
69209	114664	Hsd17b11	Mm		[43]
358	79244	Hsd17b4	Rn		[44]
	15488	Hsd17b4	Mm		[45]
	85388	FOX2	Sc	[25]	[25]
14678	14792	LPCAT3	Hs		[46]
20540	24552	Me1	Rn	[47, 48]	[4]
38298	23945	Mgll	Mm	Predicted [31]	[31]
10687	66853	Pnpla2	Mm	Predicted [31]	[31]
38109	27273	Pdk4	Mm		[49]
91131	854646	POT1	Sc	[25]	[25]
74538	20249	Scd1	Mm	[50]	[50, 51]
22223	855518	SPS19	Sc	[25]	[25]
38186	10628	TXNIP	Hs	Predicted [31]	[31, 52]
2516	22228	Ucp2	Mm		[15, 23, 53, 54]

Supplementary Material Section 5:
Expression Matrix of clustering of validation set

Mouse dataset:

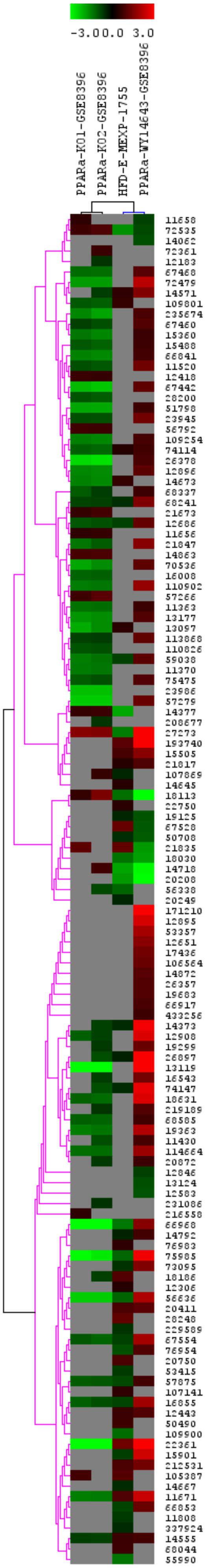

Yeast dataset:

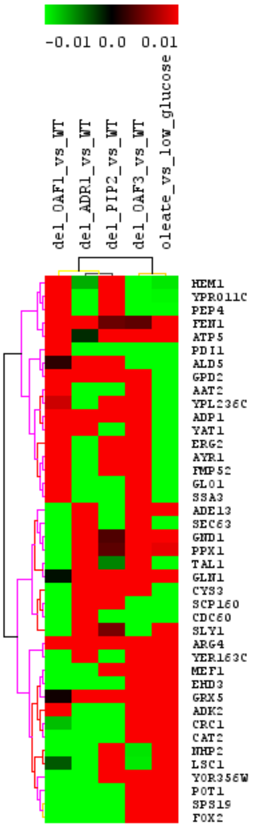

Supplementary Material Section 6: Table of Co-localized genes

Genes showing clusters distribution. In the column there are respectively the EntrezGeneID, the chromosome (C) and following the positions of start and end of the gene, the DNA strand (S) and the official symbol by HGNC. The following three columns indicate the type of gene, if a gene is identified as a target gene of PPARa, if it is a MDEG or shows functional PPRE. In the coloumn "Predicted PPRE" we can see with a diamond (♦) the target genes belonging to oPOSSUM list and with a plus (+) the target genes of the Lemay et Hwang list. In the last column we can find the GO. Genes are written in the same order of the position on chromosome, each cluster is separated from the next by a double bold line. A dotted line that separate two genes means that in the genome there are other genes that separate the two.

Entrez GeneID	C	Start	Stop	S	Official Symbol	PPARa target gene	Meta DEG	Predicted PPRE	PPRE	Gene Ontology Biological Process
14660	1	52222427	52289836	-	Gls	X [19]				GO:0006543~glutamine catabolic process*GO:0007268~synaptic transmission
227095	1	52901890	52977704	+	Hibch		X			GO:0008152~metabolic process*GO:0009083~branched chain family amino acid catabolic process*
11363	1	66877427	66909841	-	Acadl	X [3, 4]	X			GO:0006631~fatty acid metabolic process*GO:0055114~oxidation reduction*
227231	1	67169640	67277022	+	Cps1	X [19]				GO:0000050~urea cycle*GO:0006541~glutamine metabolic process
227289	1	74325174	74326163	+	Gpbar1	X [39]				GO:0007165~signal transduction*GO:0007186~G-protein coupled receptor protein signaling pathway*
104086	1	74760148	74784464	+	Cyp27a1	X [55, 56]				GO:0055114~oxidation reduction*
20872	1	75207430	75212181	+	Stk16		X			GO:0006468~protein amino acid phosphorylation*
11611	1	95031853	95041998	+	Agxt	X [19]				GO:0008152~metabolic process*GO:0046487~glyoxylate metabolic process
110611	1	95302517	95375385	-	Hdlbp		X			GO:0006629~lipid metabolic process*GO:0006869~lipid transport
15483	1	195047834	195090239	-	Hsd11b1	X [18, 42]	X			GO:0006629~lipid metabolic process*GO:0055114~oxidation reduction*
14373	1	195098354	195099286	-	G0s2	X [36]	X	+	X [36]	GO:0007049~cell cycle*
228775	2	152163161	152169796	-	Trib3	X [57]			X [57]	GO:0006355~regulation of transcription, DNA-dependent*GO:0006468~protein amino acid phosphorylation
15901	2	152562010	152563146	+	Id1		X			GO:0000122~negative regulation of transcription from RNA polymerase II promoter
11832	4	40980110	40995003	-	Aqp7	X [58]				GO:0006810~transport*GO:0006833~water transport*
18391	4	41685369	41688186	-	Oprs1		X			GO:0006810~transport*GO:0006869~lipid transport*
76238	4	44994283	45003568	+	Grhpr	X [19]			X [59]	GO:0008152~metabolic process*GO:0055114~oxidation reduction*
72535	4	45811894	45817480	+	Aldh1b1		X			GO:0008152~metabolic process*GO:0055114~oxidation reduction*
72479	4	59594463	59631566	+	Hsdl2		X			GO:0008152~metabolic process*GO:0055114~oxidation reduction*
17840	4	60510886	60514832	-	Mup1	X [60, 61]				GO:0006810~transport*
13119	4	115158750	115168737	-	Cyp4a14	X [20, 26]	X			GO:0055114~oxidation reduction*
13117	4	115190906	115206252	+	Cyp4a10	X [26]				GO:0006631~fatty acid metabolic process*GO:0055114~oxidation reduction*
114664	5	104418784	104450815	-	Hsd17b11	X [43]	X			GO:0008610~lipid biosynthetic process*GO:0055114~oxidation reduction
20750	5	104865225	104870062	+	Spp1		X			GO:0001503~ossification*GO:0006916~anti-apoptosis*GO:0007155~cell adhesion

337924	5	146585559	146617450	-	Cyp3a44		X			GO:0055114~oxidation reduction*
13112	5	146666183	146691430	-	Cyp3a11	X [26]				GO:0055114~oxidation reduction*
67442	6	72548622	72557482	+	Retsat		X			GO:0042572~retinol metabolic process*GO:0055114~oxidation reduction*
56451	6	73198667	73226876	+	Suclg1		X			GO:0006099~tricarboxylic acid cycle*GO:0008152~metabolic process*
235674	9	119057161	119066211	-	Acaa1b		X			-
113868	9	119250412	119259413	+	Acaa1a		X			GO:0006629~lipid metabolic process*GO:0006631~fatty acid metabolic process
20520	11	53678044	53705162	-	Slc22a5	X [2, 62]			X [63]	GO:0007005~mitochondrion organization*GO:0009437~carnitine metabolic process
56517	11	53764326	53793529	-	Slc22a21	X [2, 62]				GO:0006810~transport*GO:0006811~ion transport*GO:0015879~carnitine transport*
11670	11	61022247	61031916	+	Aldh3a1	X [64]				GO:0006081~cellular aldehyde metabolic process*GO:0055114~oxidation reduction*
11671	11	61058257	61080629	-	Aldh3a2		X			GO:0006081~cellular aldehyde metabolic process*GO:0055114~oxidation reduction*
20528	11	69755788	69761692	-	Slc2a4	X [64]				GO:0015758~glucose transport*GO:0042593~glucose homeostasis
11370	11	69823694	69828909	-	Acadvl	X [3]	X	+		GO:0006629~lipid metabolic process*GO:0006631~fatty acid metabolic process
69309	11	70030294	70034496	-	Slc16a13	X [2]				GO:0006810~transport*
19014	11	98013468	98054607	-	Med1	X [65]				GO:0001889~liver development*GO:0006355~regulation of transcription, DNA-dependent
23989	11	98565905	98590749	-	Med24	X [65]				GO:0006355~regulation of transcription, DNA-dependent
171210	12	85328840	85334827	+	Acot2	X [5]	X			GO:0006629~lipid metabolic process*GO:0006637~acyl-CoA metabolic process*
26897	12	85350452	85358620	+	Acot1	X [5]	X			GO:0001676~long-chain fatty acid metabolic process*GO:0006637~acyl-CoA metabolic process*
432676	12	85599197	85607161	-	EG432676		X			-
13850	14	66703214	66743337	-	Ephx2	X [66]				GO:0008152~metabolic process*GO:0019439~aromatic compound catabolic process*
110902	14	66759797	66771785	+	Chrna2		X			GO:0006810~transport*GO:0006811~ion transport*
12895	15	89246836	89255778	-	Cpt1b	X [14, 23]	X	♦		GO:0006629~lipid metabolic process*GO:0006631~fatty acid metabolic process
12651	15	89256798	89260330	-	Chkb		X			GO:0006656~phosphatidylcholine biosynthetic process*GO:0007517~muscle development*
19125	16	18071819	18089283	-	Prodh		X			GO:0006537~glutamate biosynthetic process*GO:0006560~proline metabolic process
12846	16	18407636	18426608	-	Comt1		X			GO:0006584~catecholamine metabolic process*GO:0042135~neurotransmitter catabolic process
68241	17	26000643	26005683	-	9530058B02Rik		X			GO:0008150~biological_process*
26378	17	26218156	26227109	-	Decr2		X			GO:0008152~metabolic process*GO:0055114~oxidation reduction*
27409	17	85057574	85082263	-	Abcg5	X [56]				GO:0006810~transport*
67470	17	85082471	85099673	+	Abcg8	X [56]				GO:0015918~sterol transport*GO:0042632~cholesterol homeostasis
72416	17	85104587	85190126	-	Lrpprc		X			GO:0006355~regulation of transcription, DNA-dependent*GO:0051028~mRNA transport*
13095	19	39361575	39405203	+	Cyp2c29	X [26]				GO:0055114~oxidation reduction*
13097	19	39464046	39537565	-	Cyp2c38		X			GO:0055114~oxidation reduction*
107141	19	40164186	40188441	+	Cyp2c50		X			GO:0055114~oxidation reduction*
20411	19	40366530	40588302	-	Sorbs1		X	♦		GO:0008286~insulin receptor signaling pathway*GO:0015758~glucose transport
21673	19	41103765	41134015	+	Dntt		X			GO:0006260~DNA replication*GO:0006304~DNA modification*
14718	19	43574245	43598999	-	Got1	X [19, 37]	X			GO:0006107~oxaloacetate metabolic process*GO:0006520~amino acid metabolic process
12780	19	43856798	43912708	+	Abcc2	X [67]				GO:0006810~transport*
20249	19	44468945	44482043	-	Scd1	X[50, 51]	X		X [50]	GO:0006629~lipid metabolic process*GO:0006633~fatty acid biosynthetic process

Supplementary Material Section 7: oPOSSUM Analysis Results

Selected Parameters

Conservation level:	Top 30% of conserved regions (min. conservation 60%)
Matrix match score:	75%
Upstream sequence length:	2000
Downstream sequence length:	2000
Number of genes submitted:	164
Number of genes included:	129
Number of genes excluded:	35

Target Genes:

Analyzed: 67442 74114 56792 140740 11671 23945 27273 68801 11370 110446 12846 18186 12895 208677 55990 12908 11564 110826 20411 15486 26378 59038 56636 50490 26557 76983 219189 12443 66917 11826 11808 67468 14792 51798 11363 67460 16008 109254 72479 22361 53415 13033 14571 14718 21673 20750 15505 56338 107869 20872 110611 74147 110902 19299 70536 12445 15483 68585 12686 106564 15901 14373 12583 109801 15107 107045 16543 15360 72416 15488 72535 74205 75985 68044 67528 110208 57266 56248 229589 12418 18391 14673 21847 12896 21835 11656 11430 69748 102294 18631 76954 14555 13177 56451 70804 52538 14080 57875 18113 216558 12183 66841 18030 68241 28030 109900 170439 16855 67554 57279 21351 66853 11658 22228 19363 13124 73095 50708 14062 75475 433256 53357 14667 18453 12651 212531 227095 114664 21817

Excluded: 11520 12306 13097 13101 13119 14377 14645 14863 14872 14964 17436 19125 19683 20208 20249 22750 23986 26357 26897 28200 28248 52530 66968 68337 72361 73046 105387 107141 113868 171210 193740 231086 235674 337924 432676

oPOSSUM Analysis

TF	TF Class	TF Supergroup	IC	Background gene hits	Background gene non-hits	Target gene hits	Target gene non-hits	Background TFBS hits	Background TFBS rate	Target TFBS hits	Target TFBS rate	Z-score	Fisher score
PARG-RXRA	NUCLEAR RECEPTOR	vertebrate	23.449	1206	13944	18	111	1344	0.0010	21	0.0019	14.35	1.452e-02

Analysis Table

Analysis Table:

TF: The name of the transcription factor.
Class: The class of TFs to which this TF belongs.
Supergroup: The taxonomic supergroup to which this TF belongs.
IC: The information content or specificity of this TFBS profile's position weight matrix.
Background gene hits: The number of genes in the background set for which this TFBS was predicted within the conserved non-coding regions.
Background gene non-hits: The number of genes in the background set for which this TFBS was NOT predicted within the conserved non-coding regions.
Target gene hits: The number of genes in the included target set for which this TFBS was predicted within the conserved non-coding regions.
Target gene non-hits: The number of genes in the included target set for which this TFBS was NOT predicted within the conserved non-coding regions.
Background TFBS hits: The number of times this TFBS was detected within the conserved non-coding regions of the background set of genes.
Target TFBS hits: The number of times this TFBS was detected within the conserved non-coding regions of the target set of genes.
Background TFBS rate: The rate of occurrence of this TFBS within the conserved non-coding regions of the background set of genes. The rate is equal to the number of times the site was predicted (background hits) multiplied by the width of the TFBS profile, divided by the total number of nucleotides in the conserved non-coding regions of the background gene set.
Target TFBS rate: The rate of occurrence of this TFBS within the conserved non-coding regions of the included target set of genes. The rate is equal to the number of times the site was predicted (target hits) multiplied by the width of the TFBS profile, divided by the total number of nucleotides in the conserved non-coding regions of the included target gene set.
Z-score: The likelihood that the number of TFBS nucleotides detected for the included target genes is significant as compared with the number of TFBS nucleotides detected for the background set. Z-score is expressed in units of magnitude of the standard deviation.
Fisher score: The probability that the number of hits vs. non-hits for the included target genes could have occured by random chance based on the hits vs. non-hits for the background set.

Table of target gene hits: Gene for which the TFBS is predicted

GeneID: The Entrez gene ID for which the TFBS is predicted.
Ensembl ID: The Ensembl gene ID for which the TFBS is predicted.
Chromosome: The chromosome on which the gene is located.
Strand: The chromosome strand on which the gene is located.
TSS: The transcription start site (TSS) chromosome location.
Promoter start: The start of the promoter search region chromosome location.
Promoter end: The end of the promoter search region chromosome location.
TFBS sequence: The sequence matching the given TFBS profile above the threshold match score.
TFBS start: The start of the TFBS in chromosome coordinates.
TFBS end: The end of the TFBS in chromosome coordinates.
TFBS orientation: The orientation of the TFBS sequence (assuming the gene sequence is positive).
TFBS score: The relative score for the sequence given the weight matrix profile.

Gene Symbol	Gene ID	Chr	Strand	TSS	Prom. Start	Prom. End	TFBS Seq.	TFBS Start	TFBS Rel. Start	TFBS End	TFBS Rel. End	TFBS Orient.	TFBS Score
Nrp1	18186	8	1	131245328	131243328	131247327	GTAAGGTGAAAAGGTCATAC	131243863	-1465	131243882	-1446	-1	12.600
	18186	8	1	131243743	131241743	131245742	GTAAGGTGAAAAGGTCATAC	131243863	121	131243882	140	-1	12.600
Cpt1b	12895	15	-1	89253629	89251630	89255629	ATGTAGGGAAAAGGTCACCA	89253860	-231	89253879	-250	1	10.900
Sorbs1	20411	19	-1	40430333	40428334	40432333	TTCGGGATCAGAGGTAACCT	40431016	-683	40431035	-702	1	11.280
	20411	19	-1	40431123	40429124	40433123	TTCGGGATCAGAGGTAACCT	40431016	108	40431035	89	1	11.280
	20411	19	-1	40436176	40434177	40438176	TGTGTTCTCAAAGGTCAGCG	40436278	-102	40436297	-121	-1	11.280
Ccnd1	12443	7	-1	144749220	144747221	144751220	GCTTGGATCAAGGGTCGTCC	144747930	1291	144747949	1272	-1	8.959
Mmd	67468	11	1	90065566	90063566	90067565	AAGTGGGTGAAAGTTCACCC	90066589	1024	90066608	1043	1	12.200
Lpcat3	14792	6	1	124628752	124626752	124630751	TGAGGAGCCAAAGGGCACAG	124629205	454	124629224	473	-1	12.260
Igfbp2	16008	1	1	72757699	72755699	72759698	TCAGAGGTCAAGTTTGAAGT	72759254	1556	72759273	1575	1	9.024
Stk16	20872	1	1	75094003	75092003	75096002	GTGAAGGACCATGGTCATCT	75095032	1030	75095051	1049	-1	8.633
Rtn4	68585	11	1	29618570	29616570	29620569	TACAAGGACAAAGGTTAGTA	29619420	851	29619439	870	-1	10.310
Hadh	15107	3	-1	131261198	131259199	131263198	CACTGGGTCAAAGGTCAAAG	131260522	677	131260541	658	1	18.720
Hmgcs2	15360	3	1	98365840	98363840	98367839	AACTGGGCCAAAGGTCTCAG	98365732	-108	98365751	-89	-1	10.600
Cxcl14	57266	13	-1	56306173	56304174	56308173	AGAGGGGCCAATGGTCCATG	56304765	1409	56304784	1390	1	11.220
St5	76954	7	-1	109393842	109391843	109395842	TTCAAGGGCAAAGTCCACAG	109393879	-37	109393898	-56	-1	10.790
Suclg1	56451	6	1	73178158	73176158	73180157	TTTGAGGACAGAGGTCCGAC	73179529	1372	73179548	1391	1	9.858
Fabp1	14080	6	1	71129471	71127471	71131470	ATATAGGCCATAGGTCAGTG	71129388	-83	71129407	-64	-1	10.010
	14080	6	1	71129471	71127471	71131470	CCTGAGAGCAATGGTCAACG	71129330	-141	71129349	-122	-1	10.660
Nnmt	18113	9	-1	48401562	48399563	48403562	ATCTGGGTCAAAGAGCATGT	48401436	127	48401455	108	1	9.983
Etfdh	66841	3	-1	79714645	79712646	79716645	AACTAGGGCAAAGGTCAATG	79714305	341	79714324	322	-1	11.990
	66841	3	-1	79714645	79712646	79716645	ATCTAGGTTAAAGGTCATGA	79714219	427	79714238	408	-1	13.600
Pnpla2	66853	7	1	141305884	141303884	141307883	CCTCAGGGCAAAGGTGATGG	141307663	1780	141307682	1799	-1	12.540
	66853	7	1	141306526	141304526	141308525	CCTCAGGGCAAAGGTGATGG	141307663	1138	141307682	1157	-1	12.540
	66853	7	1	141308941	141306941	141310940	CCTCAGGGCAAAGGTGATGG	141307663	-1278	141307682	-1259	-1	12.540

Table of target gene hits by oPOSSUM

Supplementary Material Section 8:

Comparing the observed number of genes shared by at least *j* studies with the confidence interval obtained through the permutational approach and choose those *j* showing a significant difference between observed and random number of gene enrichment. Finally among these *j* we selected the minimum *j* such that the ratio between the expected and observed number of genes shared was less than 10%. In our analysis the number of *j* leading to 4% of false positives was found to be equal to 6.

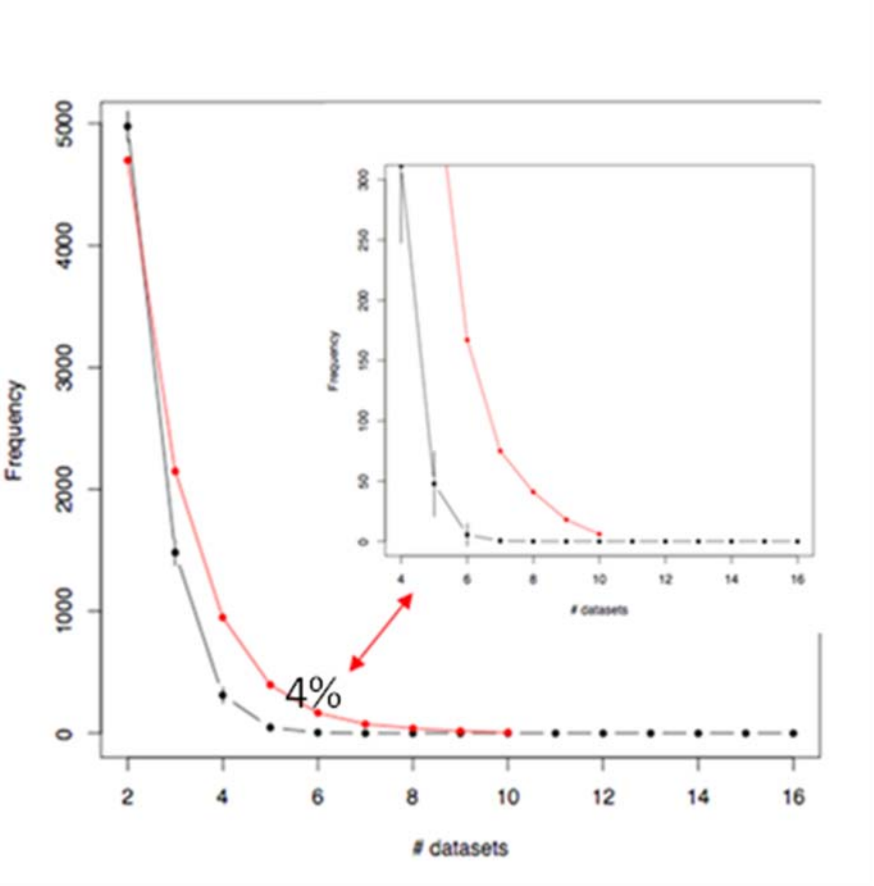

The red line represents the data, the observed.
The black line represents the simulation, the expected.
Assuming that the difference between observed and expected number of shared DEG under random selection are True Positives we can roughly calculate the percentage of False Positives as (Observed-Expected)/(Expected).

REFERENCES

1. Yamaguchi T, Matsushita S, Motojima K, Hirose F, Osumi T: MLDP, a novel PAT family protein localized to lipid droplets and enriched in the heart, is regulated by peroxisome proliferator-activated receptor alpha. *The Journal of biological chemistry* 2006, 281(20):14232-14240.
2. Hirai T, Fukui Y, Motojima K: PPARalpha agonists positively and negatively regulate the expression of several nutrient/drug transporters in mouse small intestine. *Biological & pharmaceutical bulletin* 2007, 30(11):2185-2190.
3. Aoyama T, Peters JM, Iritani N, Nakajima T, Furihata K, Hashimoto T, Gonzalez FJ: Altered constitutive expression of fatty acid-metabolizing enzymes in mice lacking the peroxisome proliferator-activated receptor alpha (PPARalpha). *The Journal of biological chemistry* 1998, 273(10):5678-5684.
4. Hashimoto T, Fujita T, Usuda N, Cook W, Qi C, Peters JM, Gonzalez FJ, Yeldandi AV, Rao MS, Reddy JK: Peroxisomal and mitochondrial fatty acid beta-oxidation in mice nullizygous for both peroxisome proliferator-activated receptor alpha and peroxisomal fatty acyl-CoA oxidase. Genotype correlation with fatty liver phenotype. *The Journal of biological chemistry* 1999, 274(27):19228-19236.
5. Hunt MC, Lindquist PJ, Peters JM, Gonzalez FJ, Diczfalusy U, Alexson SE: Involvement of the peroxisome proliferator-activated receptor alpha in regulating long-chain acyl-CoA thioesterases. *Journal of lipid research* 2000, 41(5):814-823.
6. Elholm M, Bjerking G, Knudsen J, Kristiansen K, Mandrup S: Regulatory elements in the promoter region of the rat gene encoding the acyl-CoA-binding protein. *Gene* 1996, 173(2):233-238.
7. Helledie T, Grontved L, Jensen SS, Kiilerich P, Rietveld L, Albrektsen T, Boysen MS, Nohr J, Larsen LK, Fleckner J *et al*: The gene encoding the Acyl-CoA-binding protein is activated by peroxisome proliferator-activated receptor gamma through an intronic response element functionally conserved between humans and rodents. *The Journal of biological chemistry* 2002, 277(30):26821-26830.
8. Bardot O, Aldridge TC, Latruffe N, Green S: PPAR-RXR heterodimer activates a peroxisome proliferator response element upstream of the bifunctional enzyme gene. *Biochemical and biophysical research communications* 1993, 192(1):37-45.
9. Devchand PR, Keller H, Peters JM, Vazquez M, Gonzalez FJ, Wahli W: The PPARalpha-leukotriene B4 pathway to inflammation control. *Nature* 1996, 384(6604):39-43.
10. Choudhury AI, Chahal S, Bell AR, Tomlinson SR, Roberts RA, Salter AM, Bell DR: Species differences in peroxisome proliferation; mechanisms and relevance. *Mutation research* 2000, 448(2):201-212.
11. Borst P, Elferink RO: Mammalian ABC transporters in health and disease. *Annual review of biochemistry* 2002, 71:537-592.
12. Cheema SK, Agellon LB: The murine and human cholesterol 7alpha-hydroxylase gene promoters are differentially responsive to regulation by fatty acids mediated via peroxisome proliferator-activated receptor alpha. *The Journal of biological chemistry* 2000, 275(17):12530-12536.
13. Corton JC, Bocos C, Moreno ES, Merritt A, Cattley RC, Gustafsson JA: Peroxisome proliferators alter the expression of estrogen-metabolizing enzymes. *Biochimie* 1997, 79(2-3):151-162.
14. Leone TC, Weinheimer CJ, Kelly DP: A critical role for the peroxisome proliferator-activated receptor alpha (PPARalpha) in the cellular fasting response: the PPARalpha-null mouse as a model of fatty acid oxidation disorders. *Proceedings of the National Academy of Sciences of the United States of America* 1999, 96(13):7473-7478.
15. Peters JM, Aoyama T, Burns AM, Gonzalez FJ: Bezafibrate is a dual ligand for PPARalpha and PPARbeta: studies using null mice. *Biochimica et biophysica acta* 2003, 1632(1-3):80-89.
16. Dalen KT, Ulven SM, Arntsen BM, Solaas K, Nebb HI: PPARalpha activators and fasting induce the expression of adipose differentiation-related protein in liver. *Journal of lipid research* 2006, 47(5):931-943.
17. Kersten S, Mandard S, Tan NS, Escher P, Metzger D, Chambon P, Gonzalez FJ, Desvergne B, Wahli W: Characterization of the fasting-induced adipose factor FIAF, a novel peroxisome proliferator-activated receptor target gene. *The Journal of biological chemistry* 2000, 275(37):28488-28493.
18. Yamazaki K, Kuromitsu J, Tanaka I: Microarray analysis of gene expression changes in mouse liver induced by peroxisome proliferator- activated receptor alpha agonists. *Biochemical and biophysical research communications* 2002, 290(3):1114-1122.
19. Kersten S, Mandard S, Escher P, Gonzalez FJ, Tafuri S, Desvergne B, Wahli W: The peroxisome proliferator-activated receptor alpha regulates amino acid metabolism. *Faseb J* 2001, 15(11):1971-1978.
20. Anderson SP, Yoon L, Richard EB, Dunn CS, Cattley RC, Corton JC: Delayed liver regeneration in peroxisome proliferator-activated receptor-alpha-null mice. *Hepatology (Baltimore, Md* 2002, 36(3):544-554.
21. Brandt JM, Djouadi F, Kelly DP: Fatty acids activate transcription of the muscle carnitine palmitoyltransferase I gene in cardiac myocytes via the peroxisome proliferator-activated receptor alpha. *The Journal of biological chemistry* 1998, 273(37):23786-23792.
22. Mascaro C, Acosta E, Ortiz JA, Marrero PF, Hegardt FG, Haro D: Control of human muscle-type carnitine palmitoyltransferase I gene transcription by peroxisome proliferator-activated receptor. *The Journal of biological chemistry* 1998, 273(15):8560-8563.
23. Kersten S, Seydoux J, Peters JM, Gonzalez FJ, Desvergne B, Wahli W: Peroxisome proliferator-activated receptor alpha mediates the adaptive response to fasting. *The Journal of clinical investigation* 1999, 103(11):1489-1498.
24. Barrero MJ, Camarero N, Marrero PF, Haro D: Control of human carnitine palmitoyltransferase II gene transcription by peroxisome proliferator-activated receptor through a partially conserved peroxisome proliferator-responsive element. *The Biochemical journal* 2003, 369(Pt 3):721-729.

25. Karpichev IV, Small GM: Global regulatory functions of Oaf1p and Pip2p (Oaf2p), transcription factors that regulate genes encoding peroxisomal proteins in Saccharomyces cerevisiae. *Molecular and cellular biology* 1998, 18(11):6560-6570.
26. Barclay TB, Peters JM, Sewer MB, Ferrari L, Gonzalez FJ, Morgan ET: Modulation of cytochrome P-450 gene expression in endotoxemic mice is tissue specific and peroxisome proliferator-activated receptor-alpha dependent. *The Journal of pharmacology and experimental therapeutics* 1999, 290(3):1250-1257.
27. Hunt MC, Yang YZ, Eggertsen G, Carneheim CM, Gafvels M, Einarsson C, Alexson SE: The peroxisome proliferator-activated receptor alpha (PPARalpha) regulates bile acid biosynthesis. *The Journal of biological chemistry* 2000, 275(37):28947-28953.
28. Alvares K, Fan C, Dadras SS, Yeldandi AV, Rachubinski RA, Capone JP, Subramani S, Iannaccone PM, Rao MS, Reddy JK: An upstream region of the enoyl-coenzyme A hydratase/3-hydroxyacyl-coenzyme A dehydrogenase gene directs luciferase expression in liver in response to peroxisome proliferators in transgenic mice. *Cancer research* 1994, 54(9):2303-2306.
29. Guo Y, Jolly RA, Halstead BW, Baker TK, Stutz JP, Huffman M, Calley JN, West A, Gao H, Searfoss GH *et al*: Underlying mechanisms of pharmacology and toxicity of a novel PPAR agonist revealed using rodent and canine hepatocytes. *Toxicol Sci* 2007, 96(2):294-309.
30. Jakobsson A, Jorgensen JA, Jacobsson A: Differential regulation of fatty acid elongation enzymes in brown adipocytes implies a unique role for Elovl3 during increased fatty acid oxidation. *American journal of physiology* 2005, 289(4):E517-526.
31. Rakhshandehroo M, Sanderson LM, Matilainen M, Stienstra R, Carlberg C, de Groot PJ, Muller M, Kersten S: Comprehensive Analysis of PPARalpha-Dependent Regulation of Hepatic Lipid Metabolism by Expression Profiling. *PPAR research* 2007, 2007:26839.
32. Poirier H, Niot I, Monnot MC, Braissant O, Meunier-Durmort C, Costet P, Pineau T, Wahli W, Willson TM, Besnard P: Differential involvement of peroxisome-proliferator-activated receptors alpha and delta in fibrate and fatty-acid-mediated inductions of the gene encoding liver fatty-acid-binding protein in the liver and the small intestine. *The Biochemical journal* 2001, 355(Pt 2):481-488.
33. Wolfrum C, Borrmann CM, Borchers T, Spener F: Fatty acids and hypolipidemic drugs regulate peroxisome proliferator-activated receptors alpha - and gamma-mediated gene expression via liver fatty acid binding protein: a signaling path to the nucleus. *Proceedings of the National Academy of Sciences of the United States of America* 2001, 98(5):2323-2328.
34. Oishi K, Uchida D, Ishida N: Circadian expression of FGF21 is induced by PPARalpha activation in the mouse liver. *FEBS letters* 2008, 582(25-26):3639-3642.
35. Lundasen T, Hunt MC, Nilsson LM, Sanyal S, Angelin B, Alexson SE, Rudling M: PPARalpha is a key regulator of hepatic FGF21. *Biochemical and biophysical research communications* 2007, 360(2):437-440.
36. Zandbergen F, Mandard S, Escher P, Tan NS, Patsouris D, Jatkoe T, Rojas-Caro S, Madore S, Wahli W, Tafuri S *et al*: The G0/G1 switch gene 2 is a novel PPAR target gene. *The Biochemical journal* 2005, 392(Pt 2):313-324.
37. Edgar AD, Tomkiewicz C, Costet P, Legendre C, Aggerbeck M, Bouguet J, Staels B, Guyomard C, Pineau T, Barouki R: Fenofibrate modifies transaminase gene expression via a peroxisome proliferator activated receptor alpha-dependent pathway. *Toxicology letters* 1998, 98(1-2):13-23.
38. Le May C, Pineau T, Bigot K, Kohl C, Girard J, Pegorier JP: Reduced hepatic fatty acid oxidation in fasting PPARalpha null mice is due to impaired mitochondrial hydroxymethylglutaryl-CoA synthase gene expression. *FEBS letters* 2000, 475(3):163-166.
39. Kok T, Bloks VW, Wolters H, Havinga R, Jansen PL, Staels B, Kuipers F: Peroxisome proliferator-activated receptor alpha (PPARalpha)-mediated regulation of multidrug resistance 2 (Mdr2) expression and function in mice. *The Biochemical journal* 2003, 369(Pt 3):539-547.
40. Rodriguez JC, Gil-Gomez G, Hegardt FG, Haro D: Peroxisome proliferator-activated receptor mediates induction of the mitochondrial 3-hydroxy-3-methylglutaryl-CoA synthase gene by fatty acids. *The Journal of biological chemistry* 1994, 269(29):18767-18772.
41. Hegardt FG: Transcriptional regulation of mitochondrial HMG-CoA synthase in the control of ketogenesis. *Biochimie* 1998, 80(10):803-806.
42. Hermanowski-Vosatka A, Gerhold D, Mundt SS, Loving VA, Lu M, Chen Y, Elbrecht A, Wu M, Doebber T, Kelly L *et al*: PPARalpha agonists reduce 11beta-hydroxysteroid dehydrogenase type 1 in the liver. *Biochemical and biophysical research communications* 2000, 279(2):330-336.
43. Motojima K: 17beta-hydroxysteroid dehydrogenase type 11 is a major peroxisome proliferator-activated receptor alpha-regulated gene in mouse intestine. *European journal of biochemistry / FEBS* 2004, 271(20):4141-4146.
44. Corton JC, Bocos C, Moreno ES, Merritt A, Marsman DS, Sausen PJ, Cattley RC, Gustafsson JA: Rat 17 beta-hydroxysteroid dehydrogenase type IV is a novel peroxisome proliferator-inducible gene. *Molecular pharmacology* 1996, 50(5):1157-1166.
45. Fan LQ, Cattley RC, Corton JC: Tissue-specific induction of 17 beta-hydroxysteroid dehydrogenase type IV by peroxisome proliferator chemicals is dependent on the peroxisome proliferator-activated receptor alpha. *The Journal of endocrinology* 1998, 158(2):237-246.
46. Zhao Y, Chen YQ, Bonacci TM, Bredt DS, Li S, Bensch WR, Moller DE, Kowala M, Konrad RJ, Cao G: Identification and characterization of a major liver lysophosphatidylcholine acyltransferase. *The Journal of biological chemistry* 2008, 283(13):8258-8265.
47. Castelein H, Gulick T, Declercq PE, Mannaerts GP, Moore DD, Baes MI: The peroxisome proliferator activated receptor regulates malic enzyme gene expression. *The Journal of biological chemistry* 1994, 269(43):26754-26758.

48. A J, Jeannin E, Wahli W, Desvergne B: Polarity and specific sequence requirements of peroxisome proliferator-activated receptor (PPAR)/retinoid X receptor heterodimer binding to DNA. A functional analysis of the malic enzyme gene PPAR response element. *The Journal of biological chemistry* 1997, 272(32):20108-20117.
49. Sugden MC, Bulmer K, Gibbons GF, Knight BL, Holness MJ: Peroxisome-proliferator-activated receptor-alpha (PPARalpha) deficiency leads to dysregulation of hepatic lipid and carbohydrate metabolism by fatty acids and insulin. *The Biochemical journal* 2002, 364(Pt 2):361-368.
50. Miller CW, Ntambi JM: Peroxisome proliferators induce mouse liver stearoyl-CoA desaturase 1 gene expression. *Proceedings of the National Academy of Sciences of the United States of America* 1996, 93(18):9443-9448.
51. Guillou H, Martin P, Jan S, D'Andrea S, Roulet A, Catheline D, Rioux V, Pineau T, Legrand P: Comparative effect of fenofibrate on hepatic desaturases in wild-type and peroxisome proliferator-activated receptor alpha-deficient mice. *Lipids* 2002, 37(10):981-989.
52. Billiet L, Furman C, Cuaz-Perolin C, Paumelle R, Raymondjean M, Simmet T, Rouis M: Thioredoxin-1 and its natural inhibitor, vitamin D3 up-regulated protein 1, are differentially regulated by PPARalpha in human macrophages. *Journal of molecular biology* 2008, 384(3):564-576.
53. Armstrong MB, Towle HC: Polyunsaturated fatty acids stimulate hepatic UCP-2 expression via a PPARalpha-mediated pathway. *American journal of physiology* 2001, 281(6):E1197-1204.
54. Tsuboyama-Kasaoka N, Takahashi M, Kim H, Ezaki O: Up-regulation of liver uncoupling protein-2 mRNA by either fish oil feeding or fibrate administration in mice. *Biochemical and biophysical research communications* 1999, 257(3):879-885.
55. Post SM, Duez H, Gervois PP, Staels B, Kuipers F, Princen HM: Fibrates suppress bile acid synthesis via peroxisome proliferator-activated receptor-alpha-mediated downregulation of cholesterol 7alpha-hydroxylase and sterol 27-hydroxylase expression. *Arteriosclerosis, thrombosis, and vascular biology* 2001, 21(11):1840-1845.
56. Kok T, Wolters H, Bloks VW, Havinga R, Jansen PL, Staels B, Kuipers F: Induction of hepatic ABC transporter expression is part of the PPARalpha-mediated fasting response in the mouse. *Gastroenterology* 2003, 124(1):160-171.
57. Koo SH, Satoh H, Herzig S, Lee CH, Hedrick S, Kulkarni R, Evans RM, Olefsky J, Montminy M: PGC-1 promotes insulin resistance in liver through PPAR-alpha-dependent induction of TRB-3. *Nature medicine* 2004, 10(5):530-534.
58. Walker CG, Holness MJ, Gibbons GF, Sugden MC: Fasting-induced increases in aquaporin 7 and adipose triglyceride lipase mRNA expression in adipose tissue are attenuated by peroxisome proliferator-activated receptor alpha deficiency. *International journal of obesity (2005)* 2007, 31(7):1165-1171.
59. Genolet R, Kersten S, Braissant O, Mandard S, Tan NS, Bucher P, Desvergne B, Michalik L, Wahli W: Promoter rearrangements cause species-specific hepatic regulation of the glyoxylate reductase/hydroxypyruvate reductase gene by the peroxisome proliferator-activated receptor alpha. *The Journal of biological chemistry* 2005, 280(25):24143-24152.
60. Motojima K, Passilly P, Peters JM, Gonzalez FJ, Latruffe N: Expression of putative fatty acid transporter genes are regulated by peroxisome proliferator-activated receptor alpha and gamma activators in a tissue- and inducer-specific manner. *The Journal of biological chemistry* 1998, 273(27):16710-16714.
61. Corton JC, Fan LQ, Brown S, Anderson SP, Bocos C, Cattley RC, Mode A, Gustafsson JA: Down-regulation of cytochrome P450 2C family members and positive acute-phase response gene expression by peroxisome proliferator chemicals. *Molecular pharmacology* 1998, 54(3):463-473.
62. Koch A, Konig B, Stangl GI, Eder K: PPAR alpha mediates transcriptional upregulation of novel organic cation transporters-2 and -3 and enzymes involved in hepatic carnitine synthesis. *Experimental biology and medicine (Maywood, NJ* 2008, 233(3):356-365.
63. Maeda T, Wakasawa T, Funabashi M, Fukushi A, Fujita M, Motojima K, Tamai I: Regulation of Octn2 transporter (SLC22A5) by peroxisome proliferator activated receptor alpha. *Biological & pharmaceutical bulletin* 2008, 31(6):1230-1236.
64. Mandard S, Muller M, Kersten S: Peroxisome proliferator-activated receptor alpha target genes. *Cell Mol Life Sci* 2004, 61(4):393-416.
65. Robyr D, Wolffe AP, Wahli W: Nuclear hormone receptor coregulators in action: diversity for shared tasks. *Molecular endocrinology (Baltimore, Md* 2000, 14(3):329-347.
66. Arand M, Coughtrie MW, Burchell B, Oesch F, Robertson LW: Selective induction of bilirubin UDP-glucuronosyl-transferase by perfluorodecanoic acid. *Chemico-biological interactions* 1991, 77(1):97-105.
67. Nishioka T, Hyogo H, Numata Y, Yamaguchi A, Kobuke T, Komichi D, Nonaka M, Inoue M, Nabeshima Y, Ogi M *et al*: A nuclear receptor-mediated choleretic action of fibrates is associated with enhanced canalicular membrane fluidity and transporter activity mediating bile acid-independent bile secretion. *Journal of atherosclerosis and thrombosis* 2005, 12(4):211-217.
